# Dyslipidemia in severe fever with thrombocytopenia syndrome patients: A retrospective cohort study

**DOI:** 10.1371/journal.pntd.0012673

**Published:** 2024-12-11

**Authors:** Shuai Guo, Jingliang Zhang, Qing Dong, Yunjun Yan, Chunjuan Wang, Jingyao Zhang, Lirui Tu, Shougang Guo

**Affiliations:** 1 Department of Neurology, Shandong Provincial Hospital Affiliated to Shandong First Medical University, Jinan, China; 2 Department of Neurology, Shandong Provincial Hospital, Shandong University, Jinan, China; 3 Department of Infectious Diseases, Shandong Public Health Clinical Center, Jinan, China; 4 Jinan Dian Medical Laboratory CO., LTD, Jinan, China; Australian Red Cross Lifelood, AUSTRALIA

## Abstract

**Background:**

Severe fever with thrombocytopenia syndrome (SFTS) is a rapidly progressive infectious disease triggered by a novel bunyavirus (SFTSV). Despite the critical role of host lipid metabolism in viral infections, research on dyslipidemia in SFTS remains limited.

**Methods:**

This retrospective study included 433 SFTS patients, who were stratified into survival group (n = 365) and death group (n = 68) and who were treated at the Shandong Public Health Clinical Center from September 2021 to December 2023. Additionally, 96 healthy controls with matching baseline characteristics were included from Shandong Provincial Hospital. Cross-sectional analysis based on admission data and longitudinal analysis over time were employed to survey the correlation between serum lipid profiles and mortality in SFTS patients.

**Results:**

SFTS patients exhibited elevated triglyceride (TG) levels and reduced total cholesterol (TC), high-density lipoprotein cholesterol (HDL-C), and low-density lipoprotein cholesterol (LDL-C) levels compared to healthy individuals. Cross-sectional analysis demonstrated that lower LDL-C and apolipoprotein-B (ApoB) levels were related to elevated mortality risk in SFTS patients. Longitudinal analysis demonstrated that LDL-C and ApoB levels remained consistently lower in the death group, while TG levels gradually declined, and HDL-C levels gradually increased as the disease progressed.

**Conclusion:**

SFTS patients exhibit significant dyslipidemia compared to healthy individuals. Lower LDL-C and ApoB levels may independently influence mortality in SFTS patients. Elevated TG and reduced HDL-C levels may associate with disease progression.

## Author summary

In recent years, research into Severe Fever with Thrombocytopenia Syndrome (SFTS) has intensified; however, there remains a paucity of studies addressing SFTS through the lens of serum lipid metabolism. It is well-documented that lipid metabolism plays a pivotal role in viral infections, as evidenced by numerous investigations across various viral diseases. Crucially, examining SFTS from the perspective of lipid metabolism not only enhances our understanding of viral pathogenesis but may also yield valuable insights for therapeutic interventions. This study provides a thorough examination of the serum lipid profiles in SFTS patients from a clinical perspective. Our findings first establish that SFTS patients exhibit significant lipid metabolic dysregulation compared to healthy controls. Furthermore, both cross-sectional and longitudinal analyses reveal a notable correlation between low-density lipoprotein cholesterol (LDL-C) and apolipoprotein-B (ApoB) levels and the prognosis of SFTS patients. Finally, our research opens new avenues for future investigations into SFTS by emphasizing the importance of lipid metabolism.

## 1. Introduction

Severe fever with thrombocytopenia syndrome (SFTS) is a rapidly progressive infectious disease triggered by a novel bunyavirus (SFTSV), with initially detected in Henan Province, China, in 2009 [1]. The virus is primarily transmitted by tick bites and is prevalent in hilly regions [2], with reported cases in countries such as Japan [3], Thailand [4], and South Korea [5]. According to the 2010 and 2023 editions of the Chinese National Health Commission guidelines, the death rate of untreated SFTS patients can reach 20% [6,7]. However, there are currently no specialized medications designated for the treatment of patients with SFTS.

Lipids play crucial roles in virus infection and cell-mediated immune responses, including (but not limited to) 1) their involvement in cell membrane remodeling [8–10], 2) their participation in host cell metabolism [11–13], and 3) their regulation of inflammatory responses [14–16]. During the coronavirus disease 2019 (COVID-19) pandemic, researchers analyzed the association between COVID-19 infection and disturbances in host serum lipid profiles from the perspective of lipid metabolism [17]. However, despite being discovered more than a decade ago, research on the correlation between SFTS (a viral infectious disease) and serum dyslipidemia is still limited. Only one study specifically investigating serum dyslipidemia in SFTS patients was retrieved from the *PubMed* database. A report by Huang et al. observed a notable decline in high-density lipoprotein cholesterol (HDL-C) levels upon admission in SFTS patients with poor prognosis [18]. They also investigated the relationships between SFTS and other serum lipids but did not identify any significant correlations. However, Huang et al.’s study overlooked an important source of bias in that different patients may be admitted at different times after symptom onset, thus leading to cross-sectional analysis based on admission data that would likely identify patients at different stages of the disease. Moreover, Huang et al.’s study included only 157 SFTS patients, with only 28 fatalities being reported; thus, the power of their conclusions may be insufficient.

Therefore, to further investigate the changes in serum lipid profile among SFTS patients, this larger-scale retrospective cohort study was designed to provide more robust evidence for the dyslipidemia in SFTS patients, thereby providing new perspectives on the pathogenesis and clinical treatment of SFTS. The study conformed to the guidelines of strengthening the reporting of observational studies in epidemiology (STROBE) (S1 Table) [19].

## 2. Methods

### Ethics statement

This study was approved by Ethics Review Committee of Shandong Public Health Clinical Center (No.2024XKYYEC-03) and Ethics Review Committee of Shandong Provincial Hospital (SWYX:NO. 2022–246). Given the retrospective nature of this study, informed consent from patients was waived.

### 2.1 Study design, setting, and participants

The study initially enrolled 484 hospitalized patients diagnosed with SFTS at Shandong Public Health Clinical Center between September 2021 and December 2023. After the exclusion criteria were applied, 433 patients were ultimately encompassed in the analysis. Inclusion criteria: patients who were diagnosed with SFTS by clinical physicians based on the diagnostic criteria of the Chinese National Health Commission guidelines (edition 2010 [6] and edition 2023 [7]) and who had at least one positive serum SFTSV-PCR result. Exclusion criteria: (1) hospitalized stay less than 48 hours; (2) concomitant infection with severe acute respiratory syndrome coronavirus 2 (SARS-CoV-2), hepatitis C virus, syphilis, suspected human immunodeficiency virus (HIV), epidemic hemorrhagic fever virus (EHFV), Mycobacterium tuberculosis, or Brucella; and (3) no serum lipid measurements within 24 hours of admission. SFTS patients were subsequently stratified into survival group (n = 365) and death group (n = 68).

**Fig 1:**
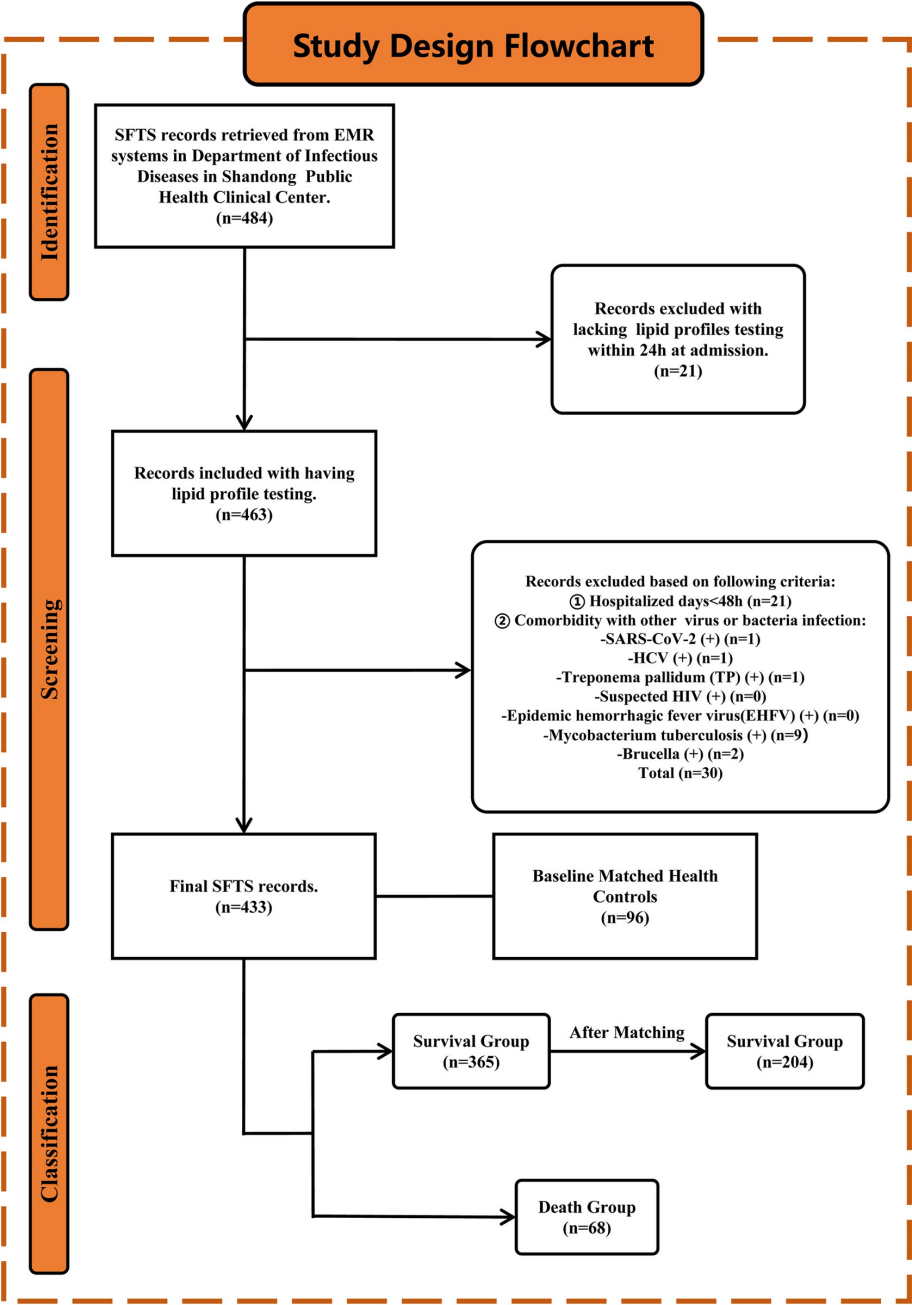
Study design flowchart. Initially, 484 patients diagnosed with SFTS were retrieved from the EMR system. A total of 433 SFTS patients were ultimately encompassed. 96 baseline-matched healthy individuals were included for comparison with SFTS patients. The SFTS patients were subsequently stratified into survival (n = 365) and death (n = 68) groups. After performing 3:1 matching, 204 data points from the survival group were retained.

Data for healthy controls were collected in June 2023 from the Center of Health Management at Shandong Provincial Hospital. A total of 96 healthy controls were selected and approximately matched for sex, age, and BMI. The detailed study design process is shown in Fig 1.

### 2.2 Variables and clinical definitions

The serum lipid profiles that were assessed in this study included triglyceride (TG), total cholesterol (TC), HDL-C, low-density lipoprotein cholesterol (LDL-C), lipoprotein(a), apolipoprotein-A1 (ApoAI), apolipoprotein-B (ApoB), and ApoAI/ApoB. The units and normal ranges for these results are listed in S2 Table. It should be noted that lipid profile measurements from health check-ups do not include lipoprotein(a), ApoAI, ApoB, or ApoAI/ApoB; therefore, these values are missing for healthy controls.

Potential confounders that may influence serum lipids included gender, age, body mass index (BMI), hypertension history, diabetes history, cerebrovascular disease history, cardiovascular disease history, chronic hepatitis B history, suspected hypothyroidism, statin history, smoking history, drinking history, and time from onset to admission.

The definitions of the variables that were included in this study are as follows. ApoAI/ApoB was defined as the value of the ApoAI divided by the value of the ApoB. BMI was calculated based on body height and weight. A history of chronic hepatitis B was defined as a positive examine result for hepatitis B virus surface antigen (HBsAg) on admission. Suspected hypothyroidism was defined as thyroid-stimulating hormone (TSH) levels on admission that exceeded the upper threshold. Statin history was defined as patients who had taken statin medication within one month before admission. Time from onset to admission was defined as the days from when patients first reported discomfort to the day of admission.

### 2.3 Data sources

Demographic information, personal and medical history, and lipid profile results were retrieved from electronic medical records (EMRs) through standardized data collection. Lipid profile results were collected from fasted blood samples taken in the morning after hospital admission. All of the patients were followed up for at least 28 days after admission. The lipid profile results for SFTS patients were obtained from the Clinical Laboratory at Shandong Public Health Clinical Center, and that of healthy controls were collected from the Center of Health Management at Shandong Provincial Hospital. Double-checking was conducted by an experienced team of physicians to ensure the precision of the data.

### 2.4 Bias

The greatest potential bias in this study arises from the fact that SFTS patients may be at different stages of the disease upon admission. To mitigate this bias, two strategies (cross-sectional and longitudinal analyses) were employed to analyze the lipid profiles of SFTS patients. Cross-sectional analysis was performed by using lipid profiles obtained within 24 hours of admission for SFTS patients, whereas longitudinal analysis utilized lipid profiles corresponding to the days after onset of symptoms. Longitudinal analysis clearly demonstrated dynamic changes in lipid profiles as the disease progressed, thus mitigating this bias.

### 2.5 Study size

Sample sizes of 211 and 53 between SFTS and healthy controls, respectively, and of 253 and 51 between survival and death groups, respectively, achieved 90% power to reject the null hypothesis of zero effect size. This calculation assumed an effect size (Cohen’s d) of 0.5 and a significance level (α) of 0.05, according to a two-sided two-sample equal-variance t-test [20]. The actual number of samples in the study far exceeded the minimum sample size that was required to achieve a moderate effect size calculation. Therefore, the conclusions are robust. The analyses were performed by using PASS (version 2021).

### 2.6 Missing data imputation

To address missing demographic data (mainly BMI), the *missForest* package was used to impute missing data [21]. The imputation results were reported based on the out-of-bag error rate of the random forest.

### 2.7 Propensity score matching

In the comparison of serum lipid profiles between SFTS survival and death groups, propensity score matching (PSM) was employed to adjust for confounders. PSM was conducted by using the *MatchIt* package in R, with a matching ratio of 3:1 for the survival *vs*. death groups, without the use of calipers for matching. Other settings followed the default specifications of the program, including the nearest neighbor matching method, propensity score calculation using logistic regression, and calculation of the average treatment effect on the treated (ATT). Standardized mean differences (SMDs) before and after matching were calculated by utilizing the *cobalt* package, with an *SMD* < 0.1 indicating a balance between the two groups in variables.

### 2.8 Subgroup analysis

In subgroup analysis, a Cox proportional hazards regression is conducted by stratifying all confounding factors to examine the relationship between serum lipids and mortality risk in different subgroups. Interaction effect analysis is used to evaluate the extent to which confounding factors influence the relationship between lipids and mortality risk. The specific analysis method for interaction effects is as follows: For a confounding factor such as Age and a serum lipid like TG, we first establish Cox regression model 1: Surv(time, status) ~ Age + TG. Next, we establish Cox regression model 2: Surv(time, status) ~ Age + TG + Age × TG. The log-likelihood values of the two models are compared using a chi-square test to obtain the p-value for the interaction. If p < 0.05, it is considered that there is a significant interaction between Age and TG; otherwise, it is considered that no interaction exists between the two factors.

### 2.9 Sensitivity analysis

Sensitivity analyses included subgroup analyses within different populations, PSM to adjust for confounders, multivariate Cox proportional hazards models, and longitudinal analyses.

### 2.10 Statistical methods

Continuous variables are shown as medians (interquartile ranges [IQRs]) and assessed by Wilcoxon rank-sum tests. Categorical variables are shown as samples (percentages) and assessed by Fisher’s exact tests. When converting continuous variables into categorical variables, "()" indicates an open interval, whereas "[]" indicates a closed interval. Cox proportional hazards regression was employed using the *survival* package. Three models were employed for multivariate Cox regression and logistic regression. Model 1 made no adjustments for any variables; Model 2 incorporated adjustments for gender, age, and BMI; and Model 3 adjusted all confounders listed in Methods 2.2. Correlation analysis was performed by Pearson tests. Subgroup analysis assessed the interactions effects between lipid profiles and confounders. The longitudinal analysis relied on the days after the onset of symptoms. P values are presented for three significant figures and marked with asterisks: *, *p* < 0.05; **, *p* < 0.01; ***, *p* < 0.001; ****, *p* < 0.0001. All of the analyses were employed by R (version 4.3.1), with two-sided *p* < 0.05 considered statistically significant.

## 3. Results

### 3.1 Characteristics of participants

The study ultimately included 529 participants, including 433 SFTS patients and 96 baseline-matched healthy controls. Basic information of participants was presented in Table 1. All of the participants had a median age of 67 (59.0–73.0) years, with 51.04% being female. The rate of cardiovascular disease history and statin history was lower in SFTS patients than in healthy controls, whereas other confounding factors were essentially matched between the two groups.

**Table 1:** Basic Characteristics of Healthy Controls *vs*. SFTS Patients.

Variables	Total (N = 529)	Health (N = 96)	SFTS (N = 433)	P value
**Clinical Characteristics**				
Gender				0.116
Male	259(48.96%)	40(41.67%)	219(50.58%)	
Female	270(51.04%)	56(58.33%)	214(49.42%)	
Age	67.0(59.0–73.0)	67.0(59.8–71.0)	67.0(58.0–73.0)	0.627
BMI	22.96(21.16–25.23)	23.12(21.83–25.19)	22.92(20.81–25.25)	0.105
Hypertension History				0.520
N	394(74.48%)	69(71.88%)	325(75.06%)	
Y	135(25.52%)	27(28.13%)	108(24.94%)	
Diabetes History				0.622
N	459(86.77%)	82(85.42%)	377(87.07%)	
Y	70(13.23%)	14(14.58%)	56(12.93%)	
Cardiovascular Disease History				0.020*
N	478(90.36%)	80(83.33%)	398(91.92%)	
Y	51(9.64%)	16(16.67%)	35(8.08%)	
Cerebrovascular Disease History				0.709
N	475(89.79%)	85(88.54%)	390(90.07%)	
Y	54(10.21%)	11(11.46%)	43(9.93%)	
Choronic Hepatitis B History				0.780
N	508(96.03%)	93(96.88%)	415(95.84%)	
Y	21(3.97%)	3(3.13%)	18(4.16%)	
Suspected Hypothyroidism				0.801
N	502(94.90%)	92(95.83%)	410(94.69%)	
Y	27(5.10%)	4(4.17%)	23(5.31%)	
Statin History				<0.001****
N	463(87.52%)	71(73.96%)	392(90.53%)	
Y	66(12.48%)	25(26.04%)	41(9.47%)	
Smoking History				0.303
N	434(82.04%)	75(78.13%)	359(82.91%)	
Y	95(17.96%)	21(21.88%)	74(17.09%)	
Drinking History				0.212
N	418(79.02%)	71(73.96%)	347(80.14%)	
Y	111(20.98%)	25(26.04%)	86(19.86%)	
**Lipid Profiles**				
TG	1.67(1.19–2.43)	1.33(0.98–1.96)	1.76(1.25–2.52)	<0.001***
Total Cholesterol	3.40(2.77–4.12)	4.23(3.65–5.38)	3.23(2.67–3.86)	<0.001****
HDL-C	0.94(0.77–1.18)	1.25(1.08–1.57)	0.90(0.73–1.05)	<0.001****
LDL-C	1.65(1.23–2.28)	2.41(2.02–3.17)	1.51(1.14–2.00)	<0.001****
Lipoprotein(a)	-	-	67.0(30.0–166.0)	-
ApoAI	-	-	0.99(0.86–1.15)	-
ApoB	-	-	0.71(0.57–0.88)	-
ApoAI/ApoB	-	-	1.41(1.12–1.78)	-

SFTS patients were further stratified into survival group (n = 365) and death group (n = 68). Basic information of SFTS patients was presented in the *Before Matching* section of Table 2. All of the SFTS patients had a median age of 67.0 (58.0–73.0) years; additionally, 49.42% were female, and the median hospitalized stay was 10.0 (7.0–13.0) days. There were notable variances in terms of gender, age, and statin history between survival *vs*. death groups (*SMD* > 0.1), whereas other confounders were relatively balanced (*SMD* < 0.1).

**Table 2:** Before and After Matching Results of SFTS Patients in Survival *vs*. Death Groups.

Variables	Before Matching					After Matching				
	Total (N = 433)	Survival (N = 365)	Death (N = 68)	P value	SMD	Total (N = 272)	Survival (N = 204)	Death (N = 68)	P value	SMD
**Clinical Characteristics**										
Gender				0.087	0.115				0.887	0.015
Male	219(50.58%)	178(48.77%)	41(60.29%)			161(59.19%)	120(58.82%)	41(60.29%)		
Female	214(49.42%)	187(51.23%)	27(39.71%)			111(40.81%)	84(41.18%)	27(39.71%)		
Age	67.0(58.0- 73.0)	66.0(57.0- 73.0)	69.5(65.75- 75.0)	<0.001 ***	0.679	70.0(65.0- 75.0)	70.0(65.0- 75.0)	69.5(65.75- 75.0)	0.980	0.082
BMI	22.92(20.81- 25.25)	22.89(20.81- 25.18)	23.19(20.88–25.38)	0.935	0.054	22.78(20.75- 25.28)	22.55(20.51- 25.20)	23.19(20.88- 25.38)	0.520	0.013
Hypertension History				0.224	0.070				0.200	0.078
N	325(75.06%)	278(76.16%)	47(69.12%)			204(75.00%)	157(76.96%)	47(69.12%)		
Y	108(24.94%)	87(23.84%)	21(30.88%)			68(25.00%)	47(23.04%)	21(30.88%)		
Diabetes History				0.693	0.021				>0.999	0.000
N	377(87.07%)	319(87.40%)	58(85.29%)			232(85.29%)	174(85.29%)	58(85.29%)		
Y	56(12.93%)	46(12.60%)	10(14.71%)			40(14.71%)	30(14.71%)	10(14.71%)		
Cardiovascular Disease History				0.048*	0.079				0.256	0.054
N	398(91.92%)	340(93.15%)	58(85.29%)			243(89.34%)	185(90.69%)	58(85.29%)		
Y	35(8.08%)	25(6.85%)	10(14.71%)			29(10.66%)	19(9.31%)	10(14.71%)		
Cerebrovascular Disease History				0.658	0.022				0.821	0.015
N	390(90.07%)	330(90.41%)	60(88.24%)			243(89.34%)	183(89.71%)	60(88.24%)		
Y	43(9.93%)	35(9.59%)	8(11.76%)			29(10.66%)	21(10.29%)	8(11.76%)		
Choronic Hepatitis B History				>0.999	0.003				0.768	0.015
N	415(95.84%)	350(95.89%)	65(95.59%)			257(94.49%)	192(94.12%)	65(95.59%)		
Y	18(4.16%)	15(4.11%)	3(4.41%)			15(5.51%)	12(5.88%)	3(4.41%)		
Suspected Hypothyroidism				0.034 *	0.063				>0.999	0.000
N	410(94.69%)	342(93.70%)	68(100.00%)			272(100.0%)	204(100.0%)	68(100.0%)		
Y	23(5.31%)	23(6.30%)	0(0.00%)			0(0.00%)	0(0.00%)	0(0.00%)		
Statin History				0.002 **	0.132				0.104	0.088
N	392(90.53%)	338(92.60%)	54(79.41%)			234(86.03%)	180(88.24%)	54(79.41%)		
Y	41(9.47%)	27(7.40%)	14(20.59%)			38(13.97%)	24(11.76%)	14(20.59%)		
Smoking History				0.385	0.041				0.860	0.015
N	359(82.91%)	305(83.56%)	54(79.41%)			219(80.51%)	165(80.88%)	54(79.41%)		
Y	74(17.09%)	60(16.44%)	14(20.59%)			53(19.49%)	39(19.12%)	14(20.59%)		
Drinking History				0.621	0.026				>0.999	0.005
N	347(80.14%)	294(80.55%)	53(77.94%)			211(77.57%)	158(77.45%)	53(77.94%)		
Y	86(19.86%)	71(19.45%)	15(22.06%)			61(22.43%)	46(22.55%)	15(22.06%)		
Time from Onset to Admission				0.438					0.777	
≤4	104(24.02%)	87(23.84%)	17(25.00%)	>0.999	0.012	72(26.47%)	55(26.96%)	17(25.00%)	0.883	0.020
(4,6]	148(34.18%)	125(34.25%)	23(33.82%)	>0.999	0.004	90(33.09%)	67(32.84%)	23(33.82%)	0.883	0.010
(6,8]	125(28.87%)	104(28.49%)	21(30.88%)	>0.999	0.024	90(33.09%)	69(33.82%)	21(30.88%)	0.883	0.029
(8,10]	22(5.08%)	17(4.66%)	5(7.35%)	0.913	0.027	15(5.51%)	10(4.90%)	5(7.35%)	0.883	0.025
>10	34(7.85%)	32(8.77%)	2(2.94%)	0.693	0.058	5(1.84%)	3(1.47%)	2(2.94%)	0.883	0.015
Length of Stay	10.0(7.0- 13.0)	10.0(8.0- 14.0)	5.0(4.0- 6.25)	<0.001 ****	0.762	10.0(6.0- 14.0)	11.0(9.0- 14.0)	5.0(4.0- 6.25)	<0.001 ****	0.939
**Lipid Profiles**										
TG	1.76(1.25- 2.52)	1.79(1.25- 2.49)	1.61(1.28- 2.55)	0.717	0.077	1.73(1.27- 2.65)	1.79(1.26- 2.67)	1.61(1.28- 2.55)	0.584	0.002
Total Cholesterol	3.23(2.67- 3.86)	3.28(2.73- 3.94)	2.93(2.54- 3.52)	0.002 **	0.426	3.18(2.64- 3.83)	3.27(2.68- 3.98)	2.93(2.54- 3.52)	0.005 **	0.419
HDL-C	0.90(0.73- 1.05)	0.90(0.75- 1.05)	0.89(0.61- 1.14)	0.304	0.091	0.90(0.71- 1.08)	0.90(0.74- 1.08)	0.89(0.61- 1.14)	0.308	0.103
LDL-C	1.51(1.14- 2.00)	1.59(1.22- 2.09)	1.15(0.91- 1.44)	<0.001 ****	1.009	1.41(1.07- 1.89)	1.51(1.16- 1.98)	1.15(0.91- 1.44)	<0.001 ****	0.869
Lipoprotein(a)	67.0(30.0- 166.0)	68.0(33.0- 167.0)	49.5(24.8- 136.5)	0.060	0.214	62.0(27.8- 160.5)	65.5(30.8- 166.0)	49.5(24.8- 136.5)	0.149	0.152
ApoAI	0.99(0.86- 1.15)	0.99(0.87- 1.16)	0.94(0.78- 1.13)	0.109	0.233	0.99(0.84- 1.17)	0.99(0.86- 1.17)	0.94(0.78- 1.13)	0.110	0.268
ApoB	0.71(0.57- 0.88)	0.74(0.59- 0.89)	0.58(0.45- 0.76)	<0.001 ****	0.675	0.68(0.55- 0.85)	0.73(0.59- 0.87)	0.58(0.45- 0.76)	<0.001 ***	0.602
ApoAI/ApoB	1.41(1.12- 1.78)	1.40(1.11- 1.72)	1.53(1.26- 2.27)	0.006 **	0.373	1.48(1.20- 1.85)	1.48(1.17- 1.76)	1.53(1.26- 2.27)	0.052	0.331

### 3.2 Cross-sectional analysis of serum lipid profiles in SFTS patients on admission

#### 3.2.1 Comparison in serum lipid profiles between healthy controls vs. SFTS patients

Univariate analysis demonstrated that compared to healthy controls, SFTS patients exhibited higher TG levels (1.76 [1.25–2.52] *vs*. 1.33 [0.98–1.96], respectively; *p* < 0.001) and lower levels of TC (3.23 [2.67–3.86] *vs*. 4.23 [3.65–5.38], respectively; *p* < 0.001), HDL-C (0.90 [0.73–1.05] *vs*. 1.25 [1.08–1.57], respectively; *p* < 0.001), and LDL-C (1.51 [1.14–2.00] *vs*. 2.41 [2.02–3.17], respectively; *p* < 0.001) (Table 1 and S1A Fig). In the multivariate logistic regression analysis, whether the unadjusted model was partially adjusted or fully adjusted, the serum lipid profiles of SFTS patients significantly differed from that of healthy controls (S3 Table). The odds ratios (ORs) were as follows in Model 3: TG (OR = 1.628 [1.259–2.185], *p* < 0.001), TC (OR = 0.225 [0.157–0.311], *P* < 0.001), HDL-C (OR = 0.005 [0.001–0.014], *p* < 0.001), and LDL-C (OR = 0.087 [0.050–0.143], *p* < 0.001). ROC curves showed that LDL-C (AUC = 0.852 [0.813–0.890]) had the most significant discriminatory power for distinguishing between healthy controls and SFTS patients (S1B Fig).

## 3.2.2 Comparison in serum lipid profiles between the SFTS survival vs. death groups

The PSM method was applied to match patients in the SFTS survival *vs*. death groups, with balance achieved in confounders in the post-matching dataset (*SMD* < 0.1). Univariate analysis of the post-matching dataset demonstrated that, compared to survival group, the death group exhibited lower levels of TC (2.93 [2.54–3.52] *vs*. 3.27 [2.68–3.98], *p* = 0.005), LDL-C (1.15 [0.91–1.44] *vs*. 1.51 [1.16–1.98], *p* < 0.001), and ApoB (0.58 [0.45–0.76] *vs*. 0.73 [0.59–0.87], *p* < 0.001), whereas there were no notable variances detected in the levels of TG, HDL-C, lipoprotein(a), or ApoAI (*p* > 0.05) (Table 2 and S2A–S2B Fig). ROC curves demonstrated that LDL-C had the most significant discriminatory power for distinguishing between the SFTS survival *vs*. death groups in both pre- and post-matching datasets (S2C–S2D Fig).

**Fig 2:**
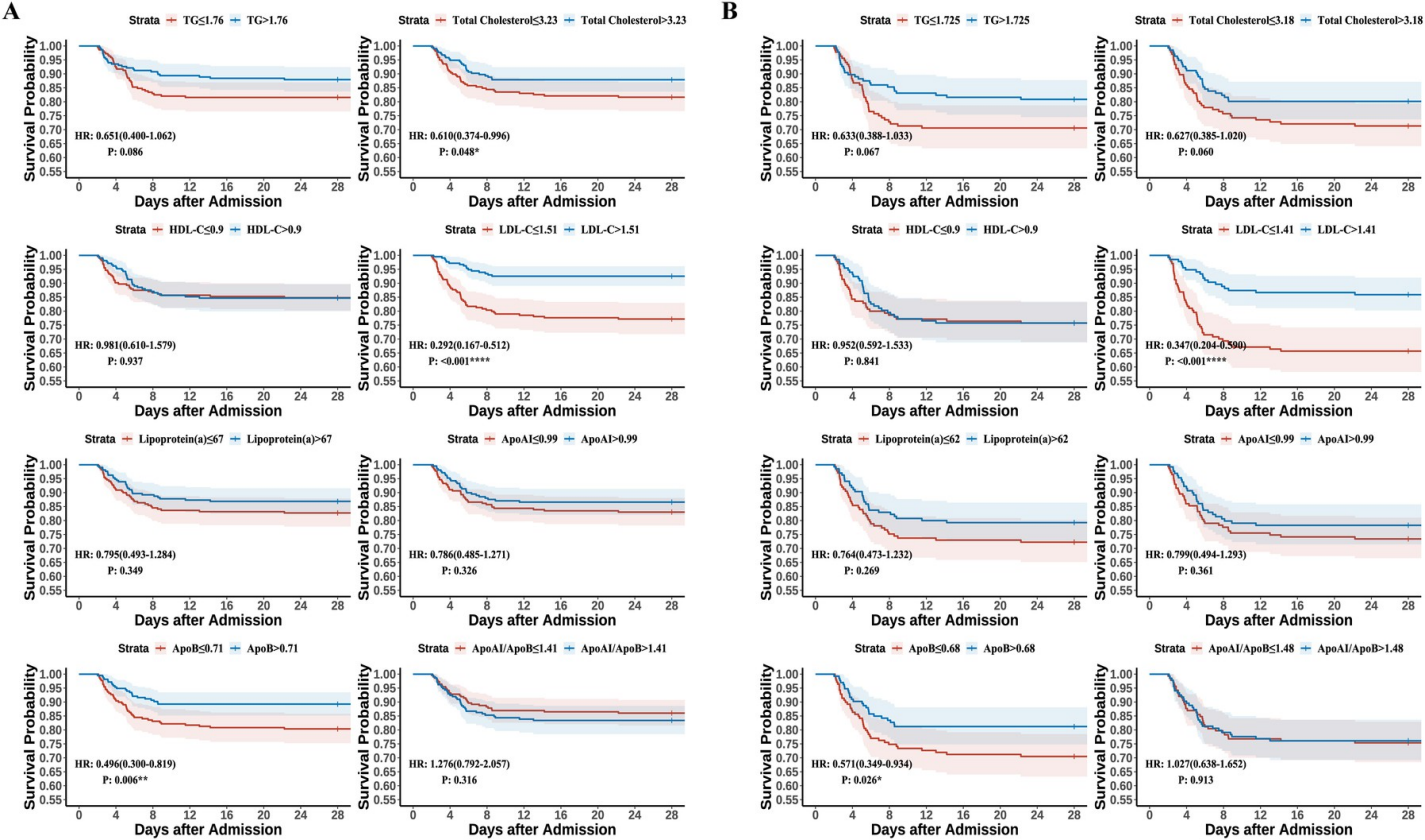
Kaplan-Meier (KM) curves for SFTS patients with varied serum lipid profiles. Survival analysis was conducted by dividing serum lipid profiles into two categories based on the median values. HR values and p values were calculated by using Cox proportional hazards regression. (a) KM curves for various serum lipid profiles in the pre-matched dataset. (b) KM curves for various serum lipid profiles in the post-matched dataset.

Multivariate Cox proportional hazards regression analysis, whether in the unadjusted, partially adjusted, or fully adjusted model in pre- or post-matching datasets, consistently demonstrated that reduced TC, LDL-C and ApoB levels, along with elevated ApoAI/ApoB ratio were related to an increased mortality risk (Table 3). In Model 3 within the post-matching dataset, the hazard ratios (HRs) were as follows: TC (HR = 0.629 [0.467–0.848], *p* = 0.002), LDL-C (HR = 0.273 [0.162–0.461], *p* <0.001), ApoB (HR = 0.088 [0.027–0.282], *p* < 0.001), and ApoAI/ApoB (HR = 1.945 [1.347–2.809], *p* < 0.001). Kaplan-Meier (KM) curves were plotted after grouping continuous variables by their median values. The KM curves in the post-matching dataset demonstrated significant differences between groups divided by LDL-C (HR = 0.347 [0.204–0.590], *p* < 0.001; LDL-C ≤ 1.41 *vs*. LDL-C > 1.41) and ApoB (HR = 0.571 [0.349–0.934], *p* = 0.026; ApoB ≤ 0.68 *vs*. ApoB > 0.68) (Fig 2).

**Table 3:** Cox Proportional Hazards Models of SFTS Patients in Survival *vs*. Death Groups.

Lipid Profiles	Model 1		Model 2		Model 3	
	HR	P value	HR	P value	HR	P value
**Before Matching**						
TG	1.060(0.904–1.242)	0.475	1.063(0.913–1.237)	0.433	1.052(0.895–1.238)	0.537
Total Cholesterol	0.634(0.473–0.851)	0.002**	0.684(0.513–0.911)	0.009**	0.663(0.495–0.886)	0.006**
HDL-C	0.634(0.256–1.566)	0.323	0.609(0.241–1.536)	0.293	0.668(0.256–1.743)	0.410
LDL-C	0.247(0.149–0.408)	<0.001****	0.288(0.176–0.472)	<0.001****	0.264(0.156–0.445)	<0.001****
Lipoprotein(a)	0.998(0.996–1.000)	0.126	0.999(0.997–1.001)	0.145	0.998(0.996–1.000)	0.123
ApoAI	0.341(0.108–1.076)	0.067	0.347(0.109–1.105)	0.073	0.373(0.113–1.238)	0.107
ApoB	0.075(0.024–0.236)	<0.001****	0.099(0.032–0.304)	<0.001****	0.088(0.027–0.284)	<0.001****
ApoAI/ApoB	1.850(1.346–2.543)	<0.001***	1.756(1.264–2.439)	<0.001***	1.846(1.308–2.606)	<0.001***
**After Matching**						
TG	1.004(0.862–1.169)	0.960	1.009(0.866–1.175)	0.910	1.000(0.844–1.185)	>0.999
Total Cholesterol	0.669(0.503–0.890)	0.006**	0.677(0.508–0.902)	0.008**	0.629(0.467–0.848)	0.002**
HDL-C	0.608(0.251–1.475)	0.272	0.596(0.241–1.472)	0.262	0.624(0.240–1.623)	0.333
LDL-C	0.308(0.187–0.508)	<0.001****	0.313(0.189–0.517)	<0.001****	0.273(0.162–0.461)	<0.001****
Lipoprotein(a)	0.999(0.997–1.001)	0.221	0.999(0.997–1.001)	0.209	0.998(0.996–1.000)	0.137
ApoAI	0.351(0.118–1.046)	0.060	0.349(0.116–1.053)	0.062	0.341(0.107–1.085)	0.069
ApoB	0.109(0.035–0.341)	<0.001***	0.113(0.036–0.355)	<0.001***	0.088(0.027–0.282)	<0.001****
ApoAI/ApoB	1.802(1.269–2.557)	<0.001***	1.791(1.254–2.558)	0.001**	1.945(1.347–2.809)	<0.001***

## 3.2.3 Subgroup analysis of serum lipid profiles and mortality risk in SFTS patients

Further subgroup analysis was applied to explore how various confounders affect the relation between serum lipid profiles and the mortality risk in SFTS patients (S4–S11 Figs). Detailed descriptions are provided in the captions of each supporting figure. The focus was particularly on the subgroup analysis of LDL-C and ApoB, which demonstrated that in most of the subgroups, there was a steady inverse relation between LDL-C and ApoB levels and the mortality risk in SFTS patients, with no significant interaction terms being identified (S7 and S10 Figs).

## 3.2.4 Correlation analysis between serum lipid profiles and the SFTSV load

Finally, the relationship between serum lipid profiles and the SFTSV load was analyzed by correlation analysis. Given that the viral load often varies exponentially by a factor of 10, the logarithm (base 10) of the SFTSV viral load was calculated. The correlation analysis of the post-matching dataset showed that TG was positively correlated with log_10_(SFTSV), whereas TC, HDL-C, LDL-C, ApoAI, and ApoB were negatively correlated with log_10_(SFTSV), and lipoprotein(a) and ApoAI/ApoB demonstrated no notable correlation with log_10_(SFTSV) (*p* > 0.05). Among these, LDL-C had the most significant correlation with log_10_(SFTSV) (*Pearson coefficient* = -0.432, *p* < 0.001) (S3 Fig and S4 Table).

### 3.3 Longitudinal analysis of serum lipid profile trends over time

**Fig 3:**
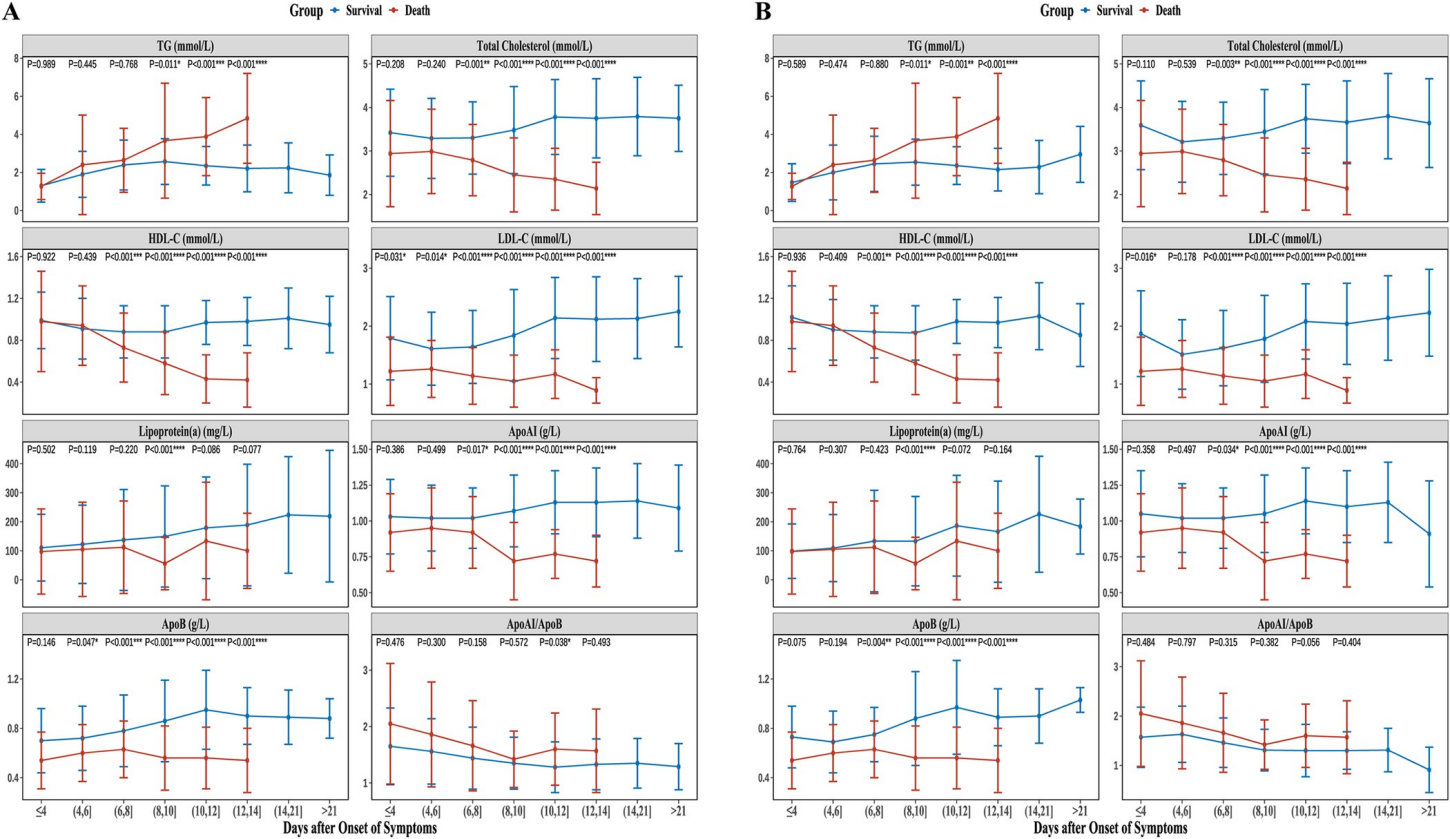
Longitudinal analysis of serum lipid profile trends over time. (a) Serum lipid profile trends over time in the pre-matched dataset. (b) Serum lipid profile trends over time in the post-matched dataset. In the figure, the x-axis "days after onset of symptoms" indicates the number of days since the patient first reported the onset of symptoms. The dynamic changes in serum lipid profiles can reveal causal relationship between serum dyslipidemia and the mortality risk in SFTS. The specific values can be found in S5 Table.

Longitudinal changes in serum lipid profiles were analyzed from the days after the onset of symptoms, categorized into eight groups: ≤4, (4,6), (6,8), (8,10), (10,12), (12,14), (14,21), and >21, with observations concluding at 14 days for patients in the death group. Detailed temporal changes in the serum lipid profile are presented in line charts (Fig 3) In the post-matching dataset, at disease onset, there were no notable variances detected in the serum TG and HDL-C levels between the survival *vs*. death groups. However, as the disease progressed, the serum TG levels gradually increased, and the HDL-C levels gradually decreased, in the death group, thus suggesting that changes in TG and HDL-C may associate with disease progression. Moreover, LDL-C and ApoB remained consistently lower in the death group from the onset of the symptoms than in the survival group, thus indicating that reduced LDL-C and ApoB levels may independently influence mortality in SFTS patients. The detailed data for each time point are presented in S5 Table.

## 4. Discussion

In this study, which ultimately included 433 SFTS patients and 96 healthy individuals, a detailed retrospective analysis of changes in serum lipid profiles was performed. Initially, comprehensive dyslipidemia was found when comparing the serum lipid profiles of SFTS patients with those of healthy individuals. Subsequently, cross-sectional analyses based on the time of admission demonstrated a close relation between lower LDL-C and ApoB levels and mortality risk in SFTS patients. Finally, the longitudinal analysis showed that TG levels gradually increased, and HDL-C levels gradually decreased, with disease progression in the death group, whereas LDL-C and ApoB remained consistently lower in the death group from the onset of the symptoms than in the survival group.

There is a notable association between viral infections and dyslipidemia, as evidenced by previous studies demonstrating significant alterations in serum lipid profiles in individuals with infections, including HIV [22], dengue virus [23], and SARS-CoV-2 [24]. In particular, during the COVID-19 pandemic, many researchers analyzed the relation between the dysregulation of host serum lipid profiles and COVID-19 from the perspective of lipid metabolism [15]. Some research demonstrated that SARS-CoV-2 infection inhibits cholesterol synthesis mediated by sterol regulatory element binding protein-2 (SREBP-2), thus clarifying why cholesterol levels decrease in patients [25]. Another study focused on the advantages of hospitalized statin medication for COVID-19 treatment; this study analyzed 13,981 patients with COVID-19 infection and confirmed that statin medication during hospitalization helps to reduce overall mortality [26]. These studies highlight the importance of investigating dyslipidemia during viral infection.

However, research on the serum dyslipidemia in SFTS patients is still limited. Our preliminary research found a close relationship between LDL-C and poor prognosis in SFTS [27]. To further explore the relationship between serum lipid dysregulation and SFTS, we searched for other studies on SFTS and lipid metabolism from *PubMed*. Only two relevant studies mentioning the correlation between blood lipids and SFTS were retrieved from the *PubMed* database. Zheng et al.’s study applied machine learning methods and reported that serum TC may be a relevant indicator for predicting the mortality of SFTS patients [28]. Huang et al. reported that lower HDL-C levels may associate with poor prognosis in SFTS patients [18]. Nonetheless, Zheng et al.’s study did not specifically focus on the correlation between serum lipid profiles and SFTS. Among the factors that they considered for analysis, only TC was selected as a lipid marker, and other lipid indicators were not included in their machine learning analysis.

Huang et al. extensively analyzed serum lipid profile changes in SFTS patients, thus confirming significant serum dyslipidemia in SFTS patients. Despite its insights, there are still several limitations in their study. First, their study had significant sources of bias, as it failed to account for the varying stages of the illness at the time of admission. According to Huang et al., the average interval from disease onset to admission was 11.2 ± 9.3 days for survivors and 10.7 ± 11.2 days for non-survivors. This timing overlaps with the typical progression of SFTS, which evolves through the following distinct stages: fever stage (3–7 days after symptoms onset), multiple organ dysfunction (MOD) stage (7–13 days after symptoms onset), and convalescence stage (11–19 days after symptoms onset) [29]. This overlap suggests that patients were at different stages of the disease upon admission, thus potentially demonstrating that the lipid comparisons were clinically irrelevant. Second, their sample size was relatively small, comprising only 157 SFTS patients (129 survivors and 28 non-survivors). With a significance level (α) of 0.05 and an effect size of 0.5, the statistical power in their test was approximately 66.4%, which is below the generally accepted minimum of 80%. This suggests that their study may be underpowered. Third, the study did not adhere to the STROBE guidelines for reporting, as it only provides box plots of lipid profiles without detailing the range, thus limiting its utility for further research. Given these issues, there is an urgent need for a more rigorously designed study that specifically focuses on serum dyslipidemia in SFTS patients, thus ensuring an adequate sample size and reduced bias.

This study addresses the aforementioned issues, thus providing strong clinical evidence of significant dyslipidemia in this disease. Due to the fact that patients who are admitted to the hospital may be at varying stages of the disease, a longitudinal analysis based on time trends is needed to further understand the patterns of change in serum lipid profiles among SFTS patients. Longitudinal analysis demonstrated that LDL-C and ApoB levels remained consistently lower in death group from the onset of the symptoms than in the survival group, indicating that reduced LDL-C and ApoB levels may independently influence mortality in SFTS patients. ApoAI and ApoB are the primary constituents of lipoproteins in HDL-C and LDL-C, respectively [30]; therefore, they generally exhibit similar trends. To further confirm this effect, additional cohort studies or Mendelian randomization studies that can eliminate reverse causation are recommended. In fact, a previous study reported of a notable relation between reduced LDL-C levels and elevated ICU admission risk in septic patients, which could be explained by comorbidities [31]. Another community-based cohort study reported that individuals with initially lower LDL-C levels exhibited an increased incidence of community-acquired sepsis, whereas no correlation with the incidence of sepsis and HDL-C levels was noted [32]. These findings imply that reduced LDL-C levels may independently heighten mortality risk in SFTS patients rather than being a result of viral infection.

It was further observed that at the onset of illness, levels of TG and HDL-C showed minimal variances between SFTS survival and death groups. However, as the disease progressed, it was observed that there were a notable elevation in TG and a marked reduction in HDL-C in the death group. This finding may explain the results of Huang et al’s study [18]. The hospital where Huang et al. conducted their study is a top-tier hospital in Jiangsu Province, China, primarily admitting critically ill patients. Therefore, the patients who were included in Huang et al.’s study were likely in the later stages of the disease, at which point HDL-C levels in the SFTS death group had already significantly decreased. Huang et al. extensively discussed the factors contributing to the decline in HDL-C levels among SFTS patients, including the inhibition of lecithin cholesterol acyltransferase activity by high levels of inflammation that reduce HDL-C synthesis [33] and the consumption of HDL-C during its involvement in anti-inflammatory processes [34]. Another notable change involves TG levels. Infection and inflammation can lead to the breakdown of adipose tissue and the production of large amounts of fatty acids. Excess fatty acids stimulate the liver to synthesize TG and secrete very low-density lipoprotein (VLDL) to transport TG [35]. Therefore, as the infection worsens, the breakdown of body fat intensifies, and the liver loses its compensatory capacity, thus resulting in a significant increase in serum TG levels and a state of hyperlipidemia, after which patients paradoxically become emaciated.

## 5. Strengths and limitations

As previously mentioned, the strengths of this study address the shortcomings of the research by Huang et al. [18]. First, an adequate sample size ensures that the conclusions have high test power. Second, the longitudinal study design eliminates the bias of SFTS patients being at different stages of the disease upon admission. Finally, strict adherence to the STROBE reporting guidelines facilitates knowledge sharing among scholars. This study provides robust evidence of serum dyslipidemia in SFTS patients and offers clinical evidence for future research on lipid metabolism in SFTS patients.

This study had certain limitations. First, all of the patient samples were obtained from a single center; therefore, the data only fully represented the situation of SFTS patients in Shandong Province, China. Second, the effect size (Cohen’s d) of 0.5 was set during the sample size calculation; thus, the results with effect sizes less than 0.5 in the cross-sectional analysis may not be adequate. Third, the imbalance in intergroup data may amplify or weaken the effects of statistical analyses. There is a sample size imbalance between the survival group and the death group (365 vs. 68), and even after matching at a 3:1 ratio, the imbalance persists (204 vs. 68). However, the degree of this data imbalance remains within an acceptable range, and its impact on the conclusions is limited.

Third, not all of the patients had lipid profile data measured at every time window during the longitudinal analysis, thus leading to potential confounders due to patient factors. A more appropriate approach would involve considering mixed-effects models for analysis; however, the complex statistical procedures and the limitations of sample size in each time window make the results less interpretable. Lastly, despite controlling for various confounding factors and employing methods to limit confounding, there may still be unquantifiable confounding factors. For example, SFTS patients are often farmers; therefore, socioeconomic status could be a potential confounding factor. Unfortunately, many SFTS fatalities voluntarily discontinued treatment for economic reasons, and it is possible that these patients could have survived if they had received adequate treatment.

## 6. Conclusion

This study comprehensively investigated the changes in the serum lipid profiles of SFTS patients, thus providing strong clinical evidence of significant dyslipidemia in this disease. In particular, longitudinal analysis indicated that lower LDL-C and ApoB levels may independently influence mortality rate of SFTS patients. These findings provide new insights into the dysregulation of lipid metabolism in SFTS.

## Supporting information

S1 Table STROBE statement—checklist of items. (PDF)

S2 Table Serum lipid profiles abbreviations, units and normal range in two hospital. (PDF)

S3 Table Logistic Regression Models of Healthy Controls vs. SFTS Patients. (PDF)

S4 Table Pearson Correlation Analysis between Lipid Profiles and log10(SFTSV). (PDF)

S5 Table Time Trend Analysis of SFTS Patients in Survival vs. Death Groups. (PDF)

S1 Fig Lipid profiles of healthy conrtols vs. SFTS patients. (PDF)

S2 Fig Differences in serum lipid profiles between the survival vs. death groups. (PDF)

S3 Fig Pearson correlation analysis between serum lipid profiles and log10(SFTSV) viral load. (PDF)

S4 Fig Subgroup analysis of TG. (PDF)

S5 Fig Subgroup analysis of Total Cholesterol. (PDF)

S6 Fig Subgroup analysis of HDL-C. (PDF)

S7 Fig Subgroup analysis of LDL-C. (PDF)

S8 Fig Subgroup analysis of lipoprotein(a). (PDF)

S9 Fig Subgroup analysis of ApoAI. (PDF)

S10 Fig Subgroup analysis of ApoB. (PDF)

S11 Fig Subgroup analysis of ApoAI/ApoB. (PDF)
