# Supplementary material for: Dyslipidemia in severe fever with thrombocytopenia syndrome patients: A retrospective cohort study
Source: PLoS Negl Trop Dis. 2024 Dec 11;18(12):e0012673. doi: 10.1371/journal.pntd.0012673 (PMC11634008; doi:10.1371/journal.pntd.0012673)
Supplement: S2 Table — (PDF) [file pntd.0012673.s002.pdf]

Table S2. Serum lipid profiles abbreviations, units and normal range in two hospital.

Full Name	Abbreviations	Units	Normal Range	
			Shandong Public Health Clinical Center	Shandong Provincial Hospital
Triglycerides	TG	mmol/L	<1.70	<1.7
Total Cholesterol	-	mmol/L	≤5.18	<6.2
High-density lipoprotein cholesterol	HDL-C	mmol/L	>1.04	1.04~1.55
Low-density lipoprotein cholesterol	LDL-C	mmol/L	<3.37	0~3.37
Lipoprotein(a)	-	mg/L	⩽300	-
Apolipoprotein-A1	ApoAI	g/L	1.20~1.60	-
Apolipoprotein-B	ApoB	g/L	0.80~1.05	-
