# Supplementary material for: Dyslipidemia in severe fever with thrombocytopenia syndrome patients: A retrospective cohort study
Source: PLoS Negl Trop Dis. 2024 Dec 11;18(12):e0012673. doi: 10.1371/journal.pntd.0012673 (PMC11634008; doi:10.1371/journal.pntd.0012673)
Supplement: S3 Table — (PDF) [file pntd.0012673.s003.pdf]

Table S3. Logistic Regression Models of Healthy Controls *vs.* SFTS Patients.

Lipid Profiles	Model 1		Model 2		Model 3	
	OR	P value	OR	P value	OR	P value
TG	1.516(1.196-1.992)	0.001**	1.580(1.237-2.090)	<0.001***	1.628(1.259-2.185)	<0.001***
Total Cholesterol	0.283(0.210-0.371)	<0.001****	0.241(0.173-0.326)	<0.001****	0.225(0.157-0.311)	<0.001****
HDL-C	0.013(0.005-0.031)	<0.001****	0.006(0.002-0.017)	<0.001****	0.005(0.001-0.014)	<0.001****
LDL-C	0.143(0.093-0.212)	<0.001****	0.099(0.059-0.157)	<0.001****	0.087(0.050-0.143)	<0.001****

Model 1 is a crude model unadjusted for any confounders. Model 2 is a model adjusted for age, gender, and BMI. Model 3 is a model adjusted for age, gender, BMI, hypertension history, diabetes history, cardiovascular disease history, cerebrovascular disease history, choronic hepatitis B history, suspected hypothyroidism, statin history, smoking history, and drinking history.
