# Supplementary material for: Dyslipidemia in severe fever with thrombocytopenia syndrome patients: A retrospective cohort study
Source: PLoS Negl Trop Dis. 2024 Dec 11;18(12):e0012673. doi: 10.1371/journal.pntd.0012673 (PMC11634008; doi:10.1371/journal.pntd.0012673)
Supplement: S4 Table — (PDF) [file pntd.0012673.s004.pdf]

Table S4. Pearson Correlation Analysis between Lipid Profiles and log10(SFTSV).

Lipid Profile	Before Matching		After Matching	
	Pearson Coefficients	P value	Pearson Coefficients	P value
TG	0.176	0.003**	0.206	0.005**
Total Cholesterol	-0.255	<0.001****	-0.249	<0.001***
HDL-C	-0.175	0.003**	-0.237	0.001**
LDL-C	-0.427	<0.001****	-0.432	<0.001****
Lipoprotein(a)	-0.129	0.031*	-0.099	0.177
ApoAI	-0.216	<0.001***	-0.268	<0.001***
ApoB	-0.264	<0.001****	-0.264	<0.001***
ApoAI/ApoB	0.155	0.009**	0.128	0.079

Before matching is the correlation analysis results using data before propensity score matching (PSM). After matching is the correlation analysis results using data after PSM.
