# Supplementary material for: Dyslipidemia in severe fever with thrombocytopenia syndrome patients: A retrospective cohort study
Source: PLoS Negl Trop Dis. 2024 Dec 11;18(12):e0012673. doi: 10.1371/journal.pntd.0012673 (PMC11634008; doi:10.1371/journal.pntd.0012673)
Supplement: S5 Table — (PDF) [file pntd.0012673.s005.pdf]

Table S5. Time Trend Analysis of SFTS Patients in Survival *vs.* Death Groups.

Days	≤4		(4,6]		(6,8]		(8,10]		(10,12]		(12,14]		(14,21]		>21	
	**Data Before Matching**															
**Lipid Profiles**	**Survival (n=43)**	**Death (n=7)**	**Survival (n=152)**	**Death (n=31)**	**Survival (n=199)**	**Death (n=41)**	**Survival (n=177)**	**Death (n=43)**	**Survival (n=162)**	**Death (n=14)**	**Survival (n=130)**	**Death (n=13)**	**Survival (n=229)**	**Death (n=0)**	**Survival (n=27)**	**Death (n=0)**
**TG**	1.08(0.84-1.41)	0.99(0.87-1.38)	1.65(1.22-2.18)	1.4(1.01-2.05)	2.12(1.59-2.77)	2.1(1.57-3.12)	2.33(1.79-3.01)	2.96(1.95-3.69)	2.16(1.65-2.93)	3.18(2.75-4.28)	1.93(1.42-2.64)	4.59(2.82-7.12)	1.9(1.33-2.76)	-	1.59(1.2-2.01)	-
	0.989		0.445		0.768		0.011*		<0.001***		<0.001****		-		-	
**Total Cholesterol**	3.44(2.91-3.82)	2.78(2.45-3.42)	3.19(2.69-3.97)	3.05(2.61-3.6)	3.27(2.68-3.92)	2.73(2.22-3.39)	3.39(2.76-4.16)	2.45(1.77-2.99)	3.7(3.18-4.34)	2.41(1.86-2.76)	3.75(3.09-4.35)	2.16(1.86-2.59)	3.84(3.11-4.34)	-	3.66(3.28-4.16)	-
	0.208		0.240		0.001**		<0.001****		<0.001****		<0.001****		-		-	
**HDL-C**	1(0.78-1.17)	1.2(0.61-1.38)	0.9(0.71-1.08)	0.95(0.78-1.19)	0.87(0.72-1.02)	0.70(0.52-0.90)	0.87(0.71-1.03)	0.57(0.4-0.76)	0.97(0.83-1.12)	0.33(0.26-0.6)	0.97(0.83-1.10)	0.34(0.26-0.60)	1.01(0.83-1.19)	-	0.96(0.86-1.11)	-
	0.922		0.439		<0.001***		<0.001****		<0.001****		<0.001****		-		-	
**LDL-C**	1.71(1.36-2.22)	1.29(0.89-1.37)	1.48(1.16-2.03)	1.33(0.98-1.49)	1.57(1.18-2.01)	1.08(0.87-1.36)	1.78(1.22-2.29)	0.97(0.79-1.23)	2.13(1.65-2.62)	1.07(0.88-1.52)	2.12(1.58-2.65)	0.89(0.75-0.95)	2.07(1.69-2.54)	-	2.1(1.8-2.64)	-
	0.031*		0.014*		<0.001****		<0.001****		<0.001****		<0.001****		-		-	
**Lipoprotein (a)**	71(18-180)	25(17-98)	66.5(34.5-160.5)	51(16.5-150)	76(35-166.5)	59(30-126)	85(36-187)	37(18-55.5)	129.5(50.8-239)	65(25-139.5)	105(50.3-250.3)	55(34-92)	168(71-311)	-	133(59-312.5)	-
	0.502		0.119		0.220		<0.001****		0.086		0.077		-		-	
**ApoAI**	0.98(0.82-1.14)	0.91(0.73-1.08)	1(0.86-1.17)	0.99(0.71-1.11)	1(0.88-1.14)	0.94(0.73-1.05)	1.06(0.9-1.25)	0.74(0.57-0.86)	1.12(0.96-1.3)	0.82(0.63-0.88)	1.1(0.96-1.29)	0.65(0.59-0.88)	1.15(0.97-1.29)	-	1.09(0.96-1.34)	-
	0.386		0.499		0.017*		<0.001****		<0.001****		<0.001****		-		-	
**ApoB**	0.66(0.57-0.84)	0.58(0.44-0.67)	0.69(0.55-0.85)	0.59(0.47-0.77)	0.76(0.6-0.92)	0.56(0.47-0.78)	0.83(0.67-1.03)	0.53(0.38-0.76)	0.93(0.77-1.09)	0.5(0.4-0.67)	0.9(0.73-1.06)	0.4(0.34-0.67)	0.89(0.74-1.04)	-	0.87(0.76-1.02)	-
	0.146		0.047*		<0.001***		<0.001****		<0.001****		<0.001****		-		-	
**ApoAI/ApoB**	1.58(1.13-1.9)	1.26(1.26-2.8)	1.48(1.17-1.77)	1.41(1.22-2.44)	1.36(1.11-1.64)	1.51(1.12-1.93)	1.29(1.08-1.58)	1.35(0.99-1.79)	1.25(1.02-1.46)	1.56(1.12-2.24)	1.23(1.05-1.55)	1.57(0.97-1.74)	1.26(1.05-1.57)	-	1.36(0.99-1.6)	-
	0.476		0.300		0.158		0.572		0.038*		0.493		-		-	
	**Data After Matching**															
**Lipid Profiles**	**Survival (n=29)**	**Death (n=7)**	**Survival (n=87)**	**Death (n=31)**	**Survival (n=115)**	**Death (n=41)**	**Survival (n=102)**	**Death (n=43)**	**Survival (n=93)**	**Death (n=14)**	**Survival (n=83)**	**Death (n=13)**	**Survival (n=125)**	**Death (n=0)**	**Survival (n=6)**	**Death (n=0)**
**TG**	1.17(0.89-1.7)	0.99(0.87-1.38)	1.59(1.23-2.39)	1.4(1.01-2.05)	2.15(1.57-2.74)	2.1(1.57-3.12)	2.32(1.73-2.96)	2.96(1.95-3.69)	2.16(1.62-3.07)	3.18(2.75-4.28)	1.89(1.38-2.64)	4.59(2.82-7.12)	1.88(1.28-2.84)	-	2.25(2.06-3.42)	-
	0.589		0.474		0.880		0.011*		0.001**		<0.001****		-		-	
**Total Cholesterol**	3.48(2.94-3.82)	2.78(2.45-3.42)	3.1(2.58-3.97)	3.05(2.61-3.6)	3.27(2.65-3.98)	2.73(2.22-3.39)	3.26(2.76-4.2)	2.45(1.77-2.99)	3.7(3.27-4.3)	2.41(1.86-2.76)	3.6(2.98-4.28)	2.16(1.86-2.59)	3.82(3.07-4.35)	-	3.37(3.07-3.97)	-
	0.110		0.539		0.003**		<0.001****		<0.001****		<0.001****		-		-	
**HDL-C**	1(0.79-1.25)	1.2(0.61-1.38)	0.91(0.72-1.07)	0.95(0.78-1.19)	0.87(0.71-1.03)	0.7(0.52-0.9)	0.89(0.68-1.03)	0.57(0.4-0.76)	1.01(0.85-1.11)	0.33(0.26-0.6)	0.96(0.82-1.09)	0.34(0.26-0.6)	1.04(0.83-1.22)	-	0.84(0.62-1.09)	-
	0.936		0.409		0.001**		<0.001****		<0.001****		<0.001****		-		-	

**LDL-C**	1.71(1.37-2.25)	1.29(0.89-1.37)	1.34(1.12-1.86)	1.33(0.98-1.49)	1.54(1.13-2.0)	1.08(0.87-1.36)	1.7(1.2-2.29)	0.97(0.79-1.23)	2.06(1.65-2.5)	1.07(0.88-1.52)	1.98(1.53-2.47)	0.89(0.75-0.95)	2.04(1.68-2.53)	-	2.02(1.73-2.64)	-
	0.016*		0.178		<0.001****		<0.001****		<0.001****		<0.001****		-		-	
**Lipoprotein (a)**	73(15-183)	25(17-98)	59(30-159)	51(16.5-150)	64(34-144.5)	59(30-126)	76.5(37.3-160.8)	37(18-55.5)	145(50-260)	65(25-139.5)	88(46-231.5)	55(34-92)	178(85-296)	-	166(136.3-249.8)	-
	0.764		0.307		0.423		<0.001****		0.072		0.164		-		-	
**ApoAI**	0.98(0.81-1.24)	0.91(0.73-1.08)	1.01(0.85-1.19)	0.99(0.71-1.11)	0.99(0.87-1.13)	0.94(0.73-1.05)	1.05(0.87-1.24)	0.74(0.57-0.86)	1.13(0.98-1.31)	0.82(0.63-0.88)	1.05(0.95-1.28)	0.65(0.59-0.88)	1.16(0.96-1.29)	-	0.9(0.64-1.17)	-
	0.358		0.497		0.034*		<0.001****		<0.001****		<0.001****		-		-	
**ApoB**	0.67(0.61-0.82)	0.58(0.44-0.67)	0.66(0.52-0.84)	0.59(0.47-0.77)	0.75(0.6-0.88)	0.56(0.47-0.78)	0.83(0.66-1.05)	0.53(0.38-0.76)	0.93(0.78-1.09)	0.5(0.4-0.67)	0.88(0.72-1.04)	0.4(0.34-0.67)	0.89(0.76-1.05)	-	1.06(1.05-1.06)	-
	0.075		0.194		0.004**		<0.001****		<0.001****		<0.001****		-		-	
**ApoAI/ApoB**	1.54(1.14-1.81)	1.26(1.26-2.8)	1.57(1.29-1.85)	1.41(1.22-2.44)	1.38(1.14-1.66)	1.51(1.12-1.93)	1.29(1.06-1.54)	1.35(0.99-1.79)	1.25(1.04-1.47)	1.56(1.12-2.24)	1.26(1.06-1.56)	1.57(0.97-1.74)	1.23(1.01-1.57)	-	0.85(0.61-1.12)	-
	0.484		0.797		0.315		0.382		0.056		0.404		-		-	

The 'Days' at the top represents the number of days since symptoms onset, with SFTS patients in death group having no samples in the days of '(14,21]' and '>21'. Wilcoxon tests were conducted between the survival *vs.* death groups at different time points.
