# Supplementary material for: Dyslipidemia in severe fever with thrombocytopenia syndrome patients: A retrospective cohort study
Source: PLoS Negl Trop Dis. 2024 Dec 11;18(12):e0012673. doi: 10.1371/journal.pntd.0012673 (PMC11634008; doi:10.1371/journal.pntd.0012673)
Supplement: S1 Fig — (PDF) [file pntd.0012673.s006.pdf]

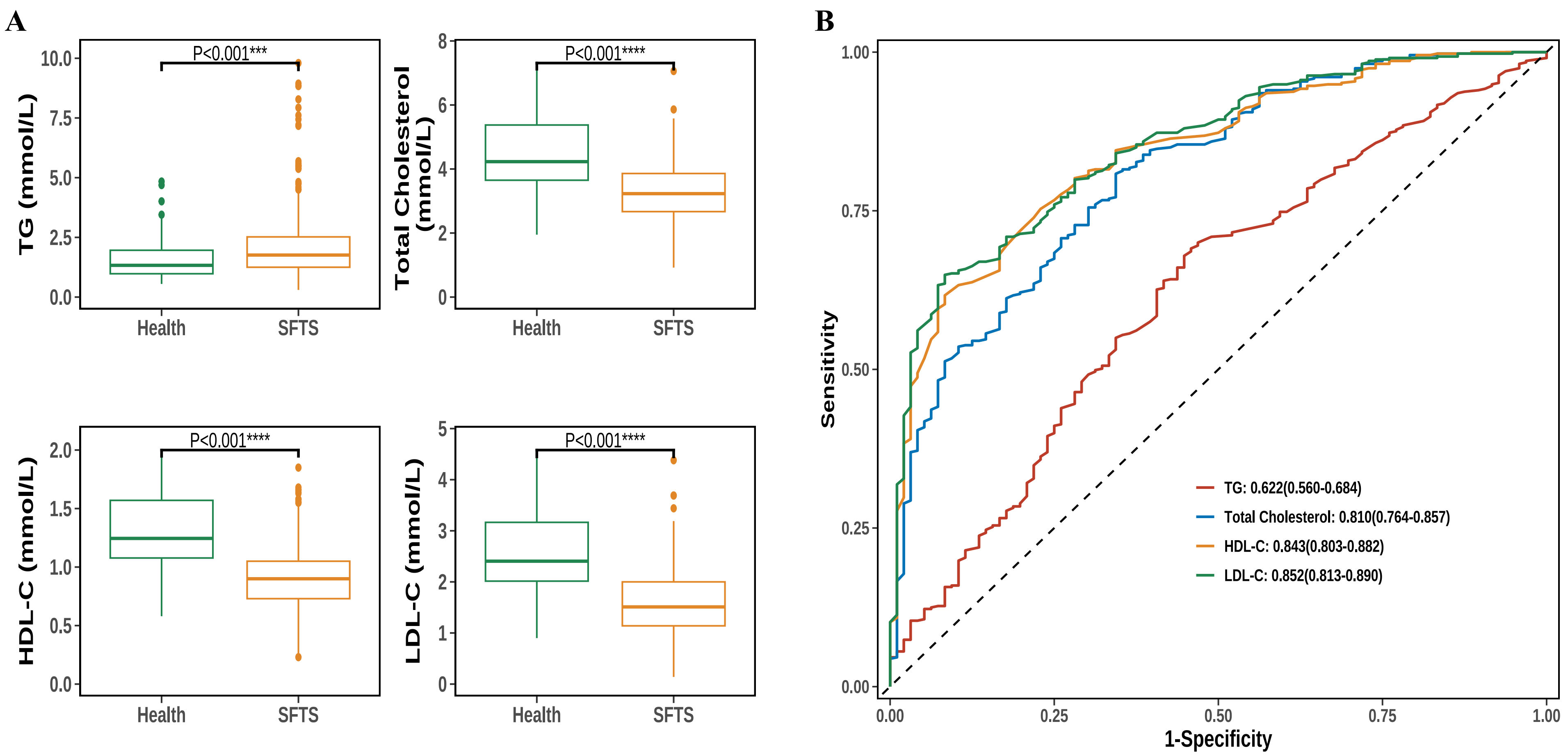

Fig S1. Lipid profiles of healthy conrtols *vs.* SFTS patients. (A) Comparison of TG, total cholesterol, HDL-C, and LDL-C between healthy controls *vs.* SFTS patients. **(B)** Logistic ROC curve of the lipid profiles between two groups. The most significant lipid differences between healthy controls *vs.* SFTS patients are LDL-C.
