# Supplementary material for: Dyslipidemia in severe fever with thrombocytopenia syndrome patients: A retrospective cohort study
Source: PLoS Negl Trop Dis. 2024 Dec 11;18(12):e0012673. doi: 10.1371/journal.pntd.0012673 (PMC11634008; doi:10.1371/journal.pntd.0012673)
Supplement: S2 Fig — (PDF) [file pntd.0012673.s007.pdf]

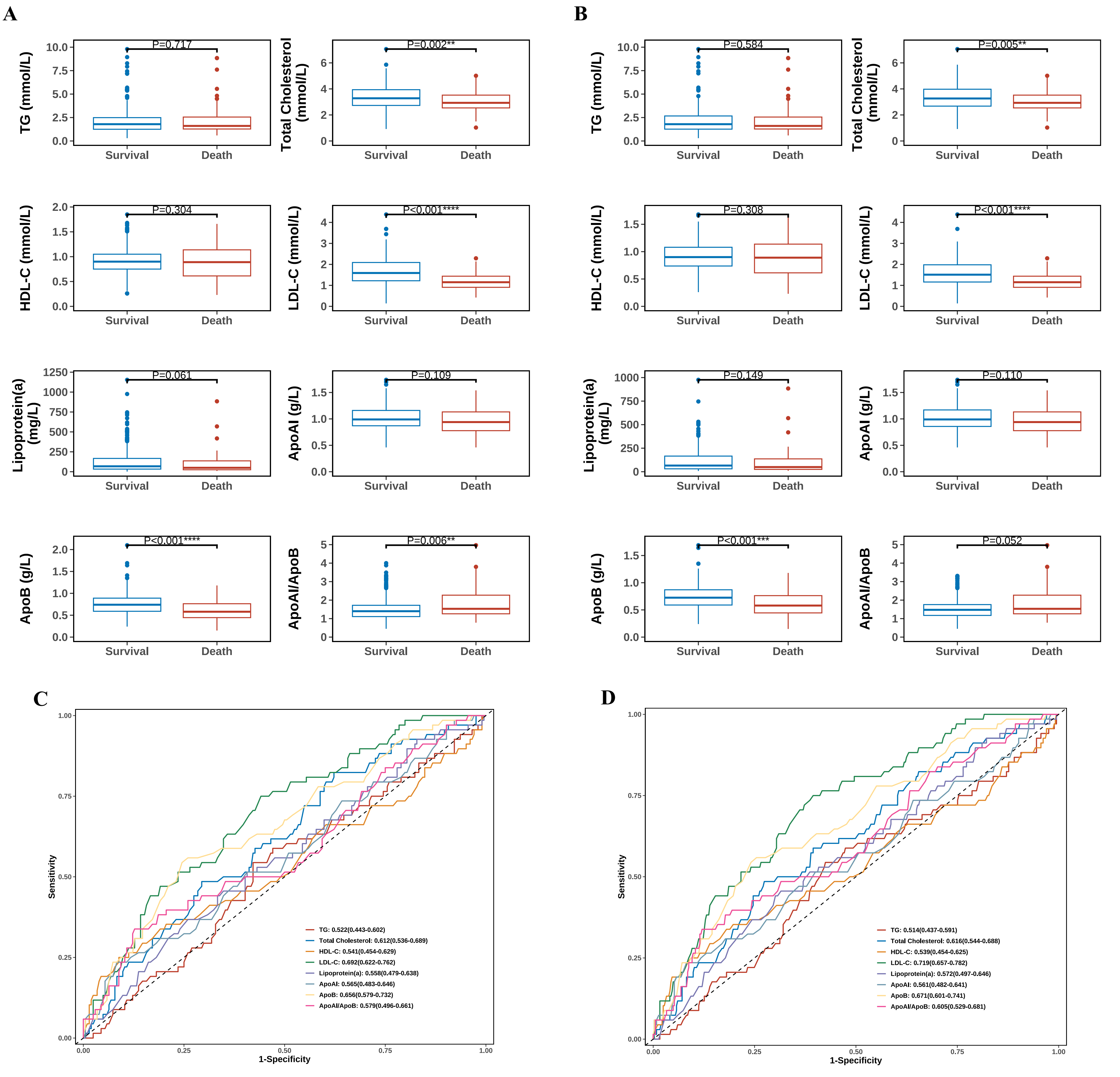

Fig S2. Differences in serum lipid profiles between the survival *vs.* death groups. (A) Differences in serum lipid profiles between the survival vs. death groups in the pre-matched dataset. **(B)** Differences in serum lipid profiles between the survival vs. death groups in the post-matched dataset. **(C)** ROC curves in the pre-matched dataset presenting the discriminative ability of different serum lipid profiles for the outcome of SFTS patients. **(D)** ROC curves in the post-matched dataset presenting the discriminative ability of different serum lipid profiles for the outcome of SFTS patients.
