# Supplementary material for: Dyslipidemia in severe fever with thrombocytopenia syndrome patients: A retrospective cohort study
Source: PLoS Negl Trop Dis. 2024 Dec 11;18(12):e0012673. doi: 10.1371/journal.pntd.0012673 (PMC11634008; doi:10.1371/journal.pntd.0012673)
Supplement: S3 Fig — (PDF) [file pntd.0012673.s008.pdf]

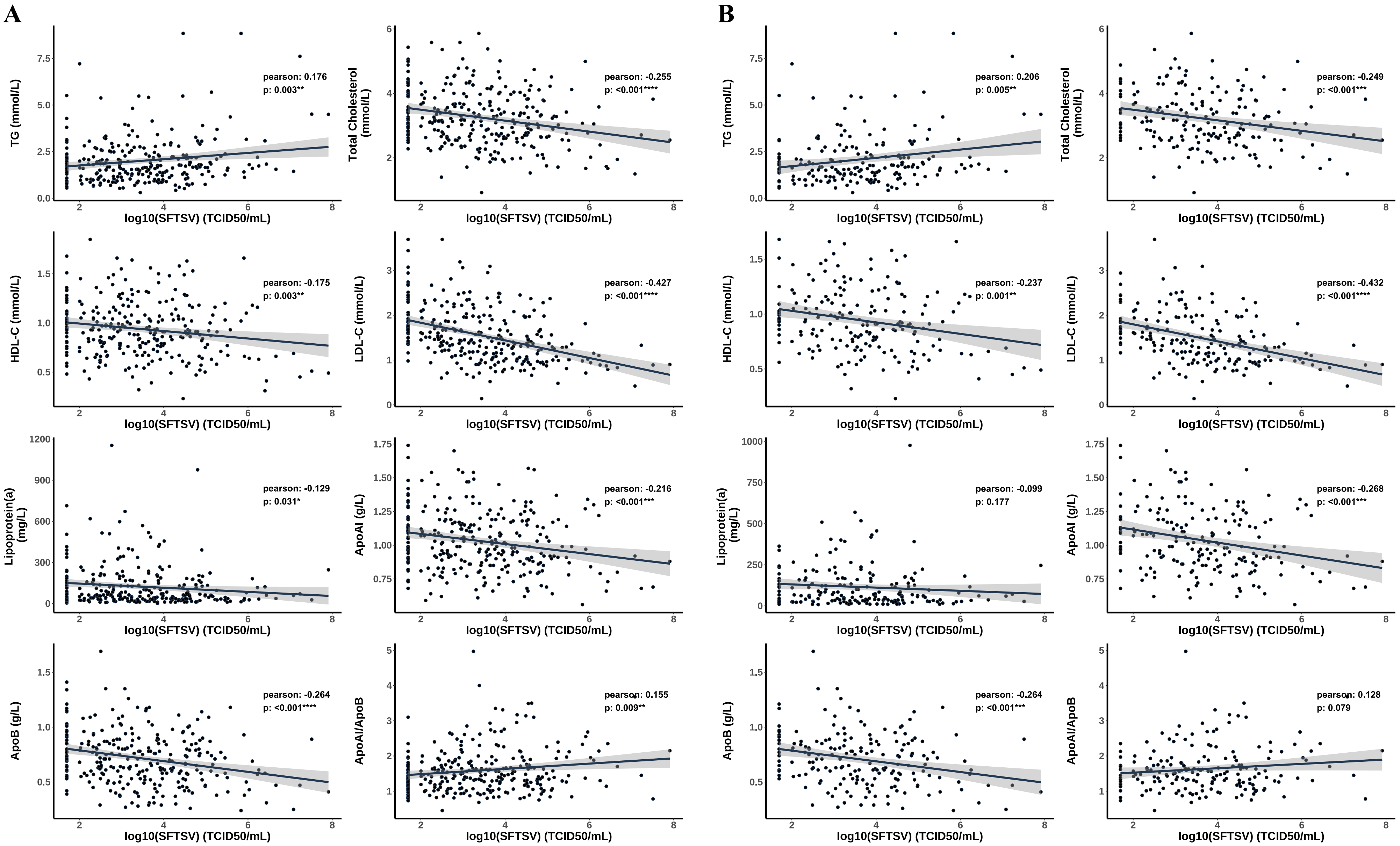

Fig S3. Pearson correlation analysis between serum lipid profiles and log10(SFTSV) viral load. The SFTSV viral load often varies exponentially by a factor of 10, thus log10 transformation was applied for correlation analysis. **(A)** Correlation analysis between serum lipid profiles and log10(SFTSV) in the pre-matched dataset. **(B)** Correlation analysis between serum lipid profiles and log10(S-FTSV) in the post-matched dataset. Specific values can be found in Table S3.
