# Supplementary material for: Dyslipidemia in severe fever with thrombocytopenia syndrome patients: A retrospective cohort study
Source: PLoS Negl Trop Dis. 2024 Dec 11;18(12):e0012673. doi: 10.1371/journal.pntd.0012673 (PMC11634008; doi:10.1371/journal.pntd.0012673)
Supplement: S4 Fig — (PDF) [file pntd.0012673.s009.pdf]

Variables	Survival N	Survival Mean	Survival SD	Death N	Death Mean	Death SD	HR per 1 SD	P value	P for Interaction
**Age**									0.134
≤60	129	2.07	1.27	7	3.41	2.67	1.651(1.077-2.532)	0.021*	
(60,65]	46	1.92	1.24	10	2.07	1.27	1.131(0.649-1.971)	0.664	
(65,70]	80	2.10	1.30	21	2.19	1.63	1.109(0.737-1.668)	0.619	
(70,75]	61	2.34	1.52	15	1.75	1.03	0.586(0.265-1.297)	0.188	
>75	49	1.94	1.39	15	2.26	2.05	1.157(0.712-1.880)	0.557	
**Gender**									0.899
Male	178	1.99	1.42	41	2.21	1.87	1.112(0.849-1.457)	0.439	
Female	187	2.17	1.24	27	2.23	1.48	1.058(0.727-1.539)	0.770	
**BMI**									0.487
≤18.5	32	1.77	0.97	7	1.68	0.98	0.951(0.430-2.101)	0.901	
(18.5,22.9]	150	2.10	1.34	25	1.98	1.19	0.893(0.582-1.369)	0.603	
(22.9,24.9]	81	1.93	0.87	17	1.84	0.80	0.873(0.515-1.479)	0.613	
>24.9	102	2.28	1.67	19	3.05	2.67	1.348(0.953-1.909)	0.092	
**Time from Onset to Admission(days)**									0.406
≤4	87	1.54	1.15	17	2.07	2.39	1.382(0.967-1.977)	0.076	
(4,6]	125	2.00	1.23	23	1.85	0.98	0.892(0.551-1.447)	0.644	
(6,8]	104	2.40	1.47	21	2.36	1.71	0.965(0.622-1.496)	0.872	
(8,10]	17	2.72	1.22	5	3.41	1.61	1.556(0.667-3.632)	0.306	
>10	32	2.55	1.26	2	3.29	1.73	3.196(0.485-21.058)	0.227	
**Hypertension History**									0.459
N	278	1.99	1.21	47	1.99	1.43	0.994(0.747-1.323)	0.968	
Y	87	2.39	1.64	21	2.73	2.19	1.203(0.813-1.780)	0.354	
**Diabetes History**									0.583
N	319	2.02	1.25	58	2.19	1.68	1.105(0.875-1.395)	0.403	
Y	46	2.52	1.76	10	2.37	2.03	0.950(0.480-1.880)	0.882	
**Cardiovascular Disease History**									0.205
N	340	2.05	1.28	58	2.24	1.81	1.136(0.903-1.430)	0.277	
Y	25	2.61	1.89	10	2.09	1.09	0.729(0.319-1.668)	0.454	
**Cerebrovascular Disease History**									0.015*
N	330	2.07	1.25	60	2.34	1.79	1.178(0.946-1.467)	0.142	
Y	35	2.23	2.00	8	1.28	0.49	0.055(0.003-1.162)	0.062	
**Choronic Hepatitis B History**									0.243
N	350	2.07	1.34	65	2.24	1.74	1.105(0.886-1.380)	0.376	
Y	15	2.47	1.24	3	1.73	1.11	0.414(0.061-2.827)	0.368	
**Suspected Hypothyroidism**									>0.999
N	342	2.11	1.37	68	2.22	1.72	1.069(0.853-1.341)	0.561	
Y	23	1.75	0.64	0					
**Smoking History**									0.068
N	305	2.17	1.41	54	2.23	1.83	1.037(0.796-1.350)	0.790	
Y	60	1.64	0.75	14	2.16	1.25	1.697(1.118-2.576)	0.013*	
**Drinking History**									0.025*
N	294	2.17	1.42	53	2.14	1.60	0.971(0.733-1.286)	0.836	
Y	71	1.75	0.85	15	2.49	2.11	1.697(1.194-2.413)	0.003**	
**Statin History**									0.658
N	338	2.04	1.24	54	2.15	1.68	1.085(0.847-1.389)	0.520	
Y	27	2.67	2.11	14	2.46	1.89	0.986(0.551-1.765)	0.962	

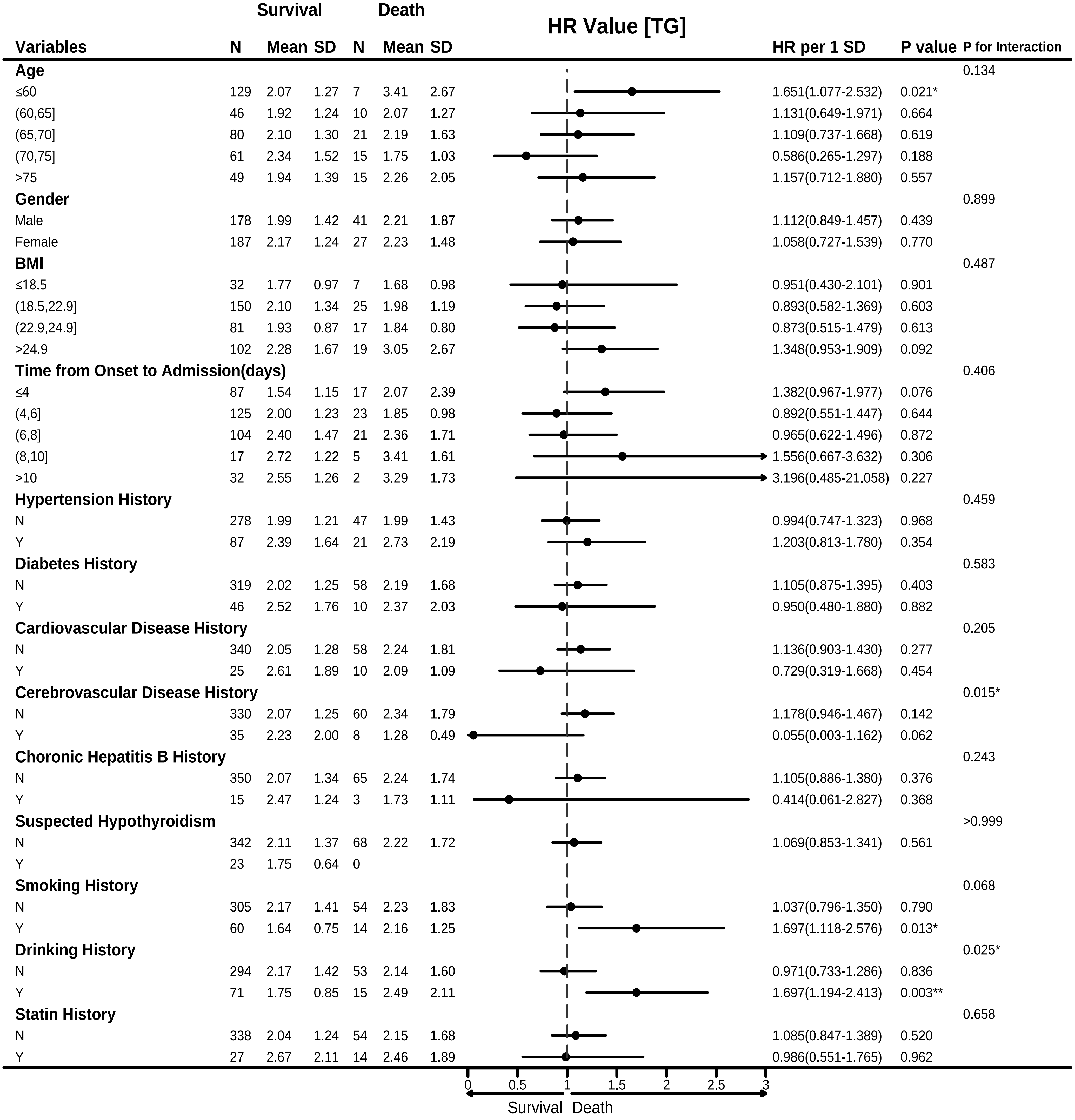

Fig S4. Subgroup analysis of TG. The degrees of interference of confounders on the relationship between serum TG and SFTS mortality are displayed in the figure. The interaction between TG and cardiovascular diseases history and drinking history is significant in the subgroup analysis.
