# Supplementary material for: Dyslipidemia in severe fever with thrombocytopenia syndrome patients: A retrospective cohort study
Source: PLoS Negl Trop Dis. 2024 Dec 11;18(12):e0012673. doi: 10.1371/journal.pntd.0012673 (PMC11634008; doi:10.1371/journal.pntd.0012673)
Supplement: S5 Fig — (PDF) [file pntd.0012673.s010.pdf]

HR Value [Total Cholesterol]

Variables	Survival N	Survival Mean	Survival SD	Death N	Death Mean	Death SD	HR per 1 SD	P value	P for Interaction
**Age**									0.765
≤60	129	3.36	0.83	7	2.95	0.43	0.587(0.259-1.327)	0.201	
(60,65]	46	3.41	0.82	10	3.13	0.98	0.716(0.371-1.381)	0.319	
(65,70]	80	3.35	0.84	21	2.85	0.82	0.595(0.380-0.929)	0.022*	
(70,75]	61	3.32	0.85	15	3.03	0.74	0.700(0.403-1.217)	0.206	
>75	49	3.23	1.02	15	3.10	0.94	0.873(0.509-1.496)	0.621	
**Gender**									0.254
Male	178	3.28	0.80	41	3.08	0.79	0.788(0.576-1.078)	0.136	
Female	187	3.40	0.91	27	2.86	0.83	0.553(0.359-0.853)	0.007**	
**BMI**									0.458
≤18.5	32	3.16	0.76	7	3.22	0.86	1.090(0.514-2.311)	0.823	
(18.5,22.9]	150	3.46	0.81	25	3.25	0.73	0.773(0.516-1.158)	0.212	
(22.9,24.9]	81	3.24	0.78	17	2.72	0.90	0.509(0.298-0.868)	0.013*	
>24.9	102	3.30	1.01	19	2.82	0.74	0.601(0.362-0.997)	0.049*	
**Time from Onset to Admission(days)**									0.732
≤4	87	3.38	0.98	17	3.00	0.86	0.654(0.378-1.133)	0.130	
(4,6]	125	3.31	0.84	23	3.16	0.78	0.838(0.555-1.267)	0.403	
(6,8]	104	3.21	0.79	21	2.86	0.87	0.665(0.428-1.032)	0.069	
(8,10]	17	3.61	1.03	5	2.69	0.61	0.250(0.052-1.197)	0.083	
>10	32	3.62	0.62	2	3.21	0.87	1.468(0.179-12.009)	0.720	
**Hypertension History**									0.337
N	278	3.36	0.82	47	3.11	0.81	0.749(0.557-1.008)	0.056	
Y	87	3.28	0.99	21	2.73	0.76	0.534(0.327-0.873)	0.012*	
**Diabetes History**									0.281
N	319	3.40	0.85	58	3.09	0.81	0.705(0.538-0.924)	0.011*	
Y	46	2.94	0.81	10	2.42	0.51	0.451(0.195-1.041)	0.062	
**Cardiovascular Disease History**									0.750
N	340	3.31	0.84	58	2.98	0.83	0.680(0.519-0.890)	0.005**	
Y	25	3.70	1.09	10	3.09	0.70	0.564(0.252-1.260)	0.163	
**Cerebrovascular Disease History**									0.237
N	330	3.36	0.86	60	3.07	0.81	0.713(0.545-0.932)	0.013*	
Y	35	3.10	0.88	8	2.42	0.60	0.428(0.185-0.990)	0.047*	
**Choronic Hepatitis B History**									0.377
N	350	3.35	0.87	65	3.01	0.82	0.684(0.528-0.886)	0.004**	
Y	15	3.10	0.66	3	2.62	0.02	0.421(0.097-1.827)	0.248	
**Suspected Hypothyroidism**									>0.999
N	342	3.34	0.86	68	2.99	0.81	0.681(0.529-0.877)	0.003**	
Y	23	1.75	0.64	0					
**Smoking History**									0.810
N	305	3.36	0.89	54	3.01	0.83	0.680(0.510-0.905)	0.008**	
Y	60	3.21	0.69	14	2.95	0.77	0.710(0.414-1.218)	0.214	
**Drinking History**									0.575
N	294	3.38	0.89	53	3.04	0.85	0.692(0.517-0.927)	0.014*	
Y	71	3.17	0.70	15	2.83	0.65	0.666(0.415-1.068)	0.092	
**Statin History**									0.719
N	338	3.33	0.82	54	3.05	0.83	0.721(0.547-0.949)	0.020*	
Y	27	3.45	1.27	14	2.76	0.69	0.575(0.281-1.177)	0.130	

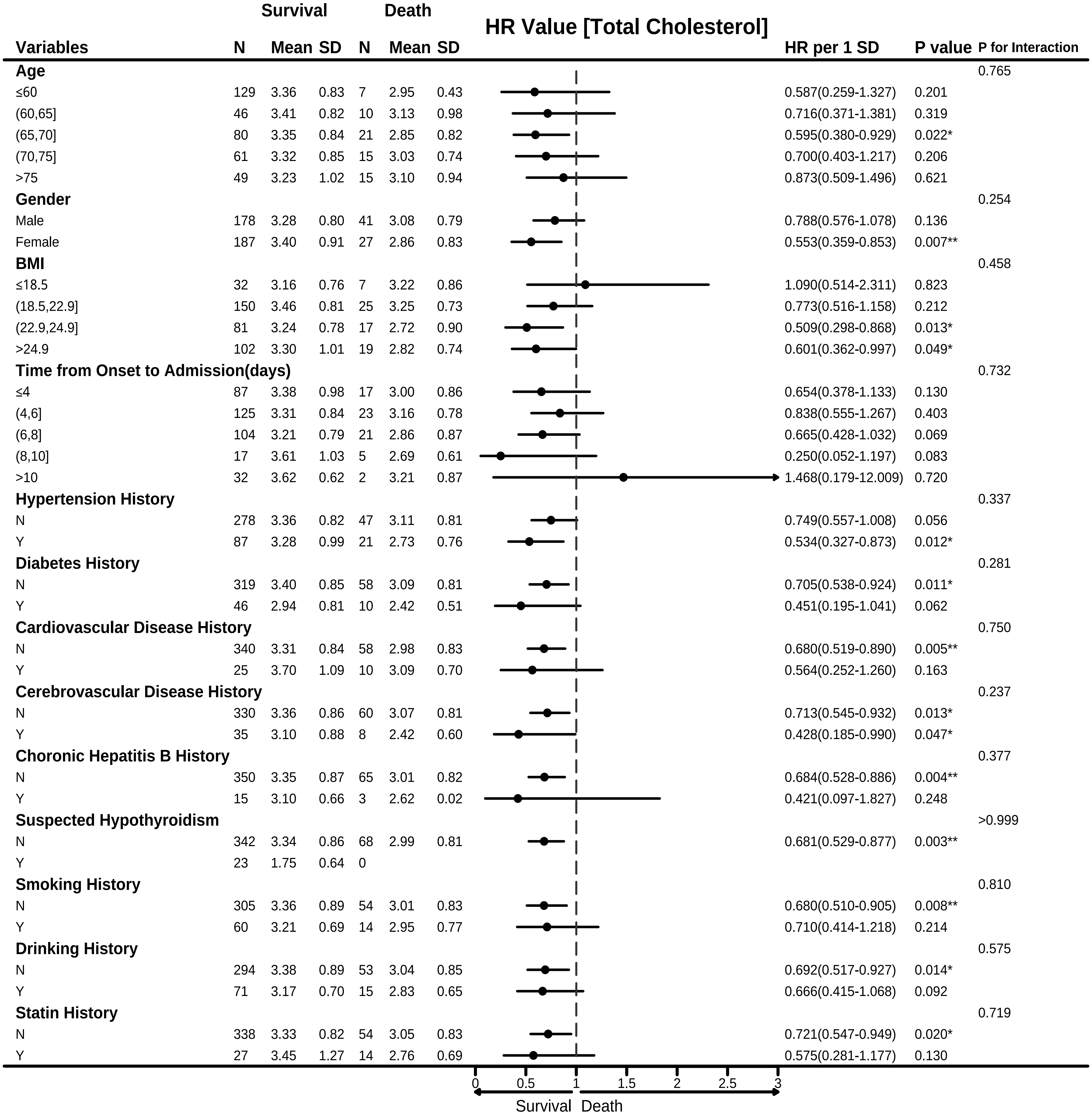

Fig S5. Subgroup analysis of Total Cholesterol. The degrees of interference of confounders on the relationship between serum total cholesterol and SFTS mortality are displayed in the figure. There are no siignificant interaction factors with total cholesterol.
