# Supplementary material for: Dyslipidemia in severe fever with thrombocytopenia syndrome patients: A retrospective cohort study
Source: PLoS Negl Trop Dis. 2024 Dec 11;18(12):e0012673. doi: 10.1371/journal.pntd.0012673 (PMC11634008; doi:10.1371/journal.pntd.0012673)
Supplement: S6 Fig — (PDF) [file pntd.0012673.s011.pdf]

Variables	Survival N	Survival Mean	Survival SD	Death N	Death Mean	Death SD	HR per 1 SD	P value	P for Interaction
**Age**									0.492
≤60	129	0.90	0.27	7	0.93	0.28	1.144(0.561-2.331)	0.711	
(60,65]	46	0.96	0.28	10	0.85	0.44	0.662(0.315-1.392)	0.276	
(65,70]	80	0.90	0.27	21	0.84	0.32	0.777(0.500-1.206)	0.261	
(70,75]	61	0.90	0.22	15	0.97	0.31	1.220(0.751-1.982)	0.422	
>75	49	0.95	0.29	15	0.85	0.40	0.721(0.417-1.246)	0.241	
**Gender**									0.306
Male	178	0.91	0.25	41	0.91	0.37	0.967(0.703-1.332)	0.839	
Female	187	0.91	0.27	27	0.84	0.32	0.752(0.498-1.134)	0.174	
**BMI**									0.061
≤18.5	32	0.98	0.29	7	1.13	0.33	1.554(0.733-3.297)	0.251	
(18.5,22.9]	150	0.96	0.28	25	0.98	0.36	1.059(0.710-1.578)	0.780	
(22.9,24.9]	81	0.89	0.24	17	0.82	0.32	0.712(0.421-1.205)	0.206	
>24.9	102	0.85	0.23	19	0.73	0.28	0.574(0.351-0.939)	0.027*	
**Time from Onset to Admission(days)**									0.141
≤4	87	0.98	0.29	17	0.98	0.42	0.969(0.583-1.609)	0.902	
(4,6]	125	0.90	0.27	23	0.89	0.33	0.959(0.634-1.452)	0.843	
(6,8]	104	0.89	0.24	21	0.87	0.33	0.955(0.610-1.495)	0.840	
(8,10]	17	0.87	0.21	5	0.61	0.10	0.216(0.048-0.979)	0.047*	
>10	32	0.92	0.23	2	0.77	0.37	0.239(0.040-1.437)	0.118	
**Hypertension History**									0.007**
N	278	0.94	0.27	47	0.97	0.35	1.093(0.820-1.457)	0.545	
Y	87	0.83	0.23	21	0.69	0.26	0.527(0.333-0.836)	0.006**	
**Diabetes History**									0.631
N	319	0.93	0.26	58	0.91	0.36	0.906(0.689-1.191)	0.478	
Y	46	0.80	0.27	10	0.73	0.24	0.770(0.397-1.494)	0.439	
**Cardiovascular Disease History**									0.697
N	340	0.91	0.26	58	0.89	0.35	0.912(0.695-1.195)	0.503	
Y	25	0.91	0.28	10	0.82	0.32	0.799(0.411-1.555)	0.510	
**Cerebrovascular Disease History**									0.577
N	330	0.92	0.27	60	0.88	0.35	0.858(0.656-1.120)	0.260	
Y	35	0.82	0.23	8	0.87	0.33	1.031(0.475-2.236)	0.938	
**Choronic Hepatitis B History**									0.661
N	350	0.92	0.27	65	0.88	0.35	0.872(0.674-1.128)	0.296	
Y	15	0.82	0.18	3	0.88	0.42	1.190(0.397-3.570)	0.756	
**Suspected Hypothyroidism**									>0.999
N	342	0.91	0.26	68	0.88	0.35	0.893(0.695-1.148)	0.378	
Y	23	1.75	0.64	0					
**Smoking History**									0.061
N	305	0.91	0.27	54	0.91	0.35	0.974(0.739-1.283)	0.849	
Y	60	0.92	0.23	14	0.79	0.35	0.533(0.280-1.016)	0.056	
**Drinking History**									0.010**
N	294	0.92	0.27	53	0.93	0.33	1.034(0.786-1.359)	0.813	
Y	71	0.89	0.23	15	0.72	0.36	0.497(0.291-0.847)	0.010*	
**Statin History**									0.244
N	338	0.92	0.26	54	0.92	0.37	0.992(0.753-1.307)	0.953	
Y	27	0.81	0.28	14	0.73	0.18	0.671(0.394-1.145)	0.143	

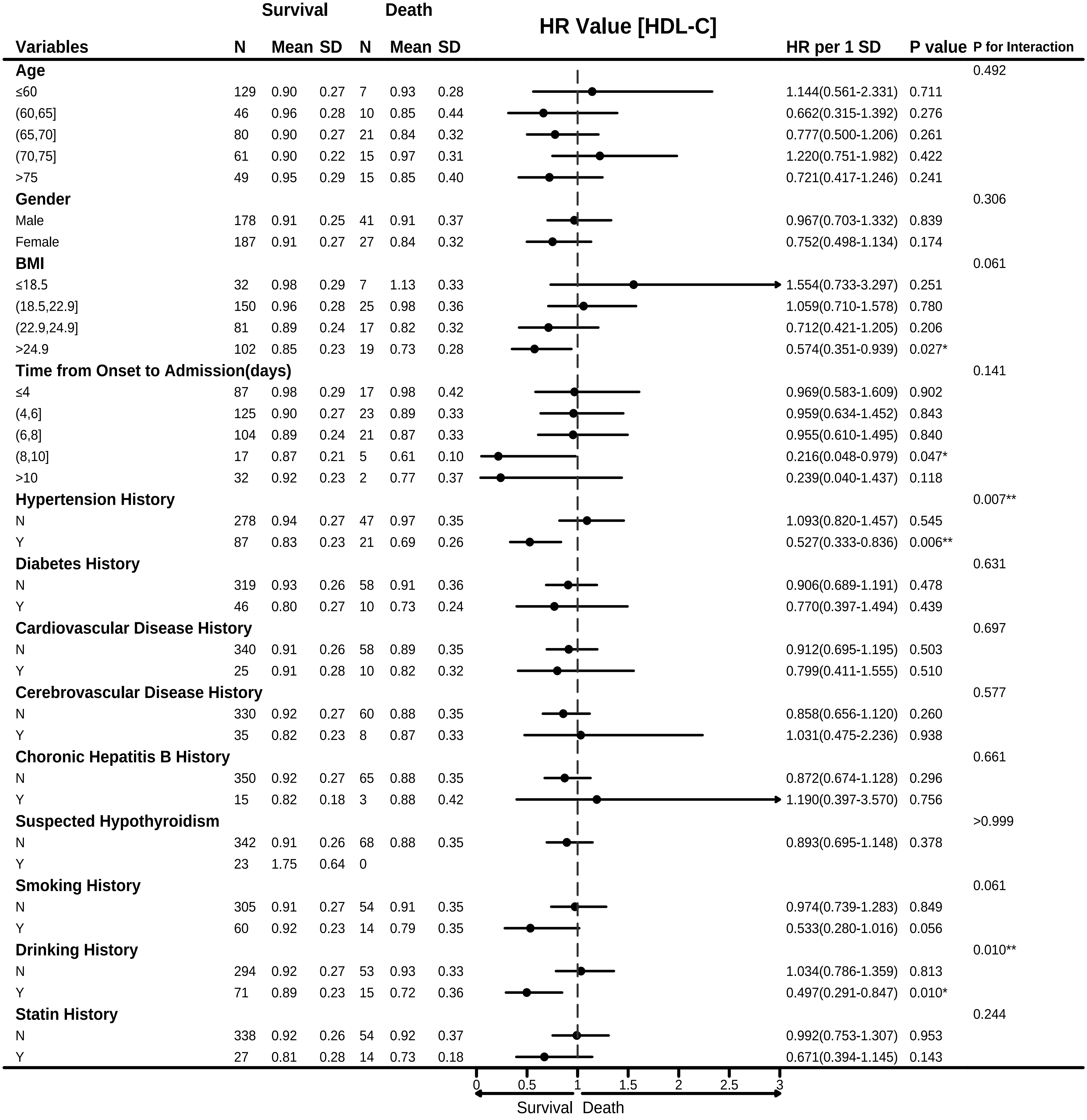

Fig S6. Subgroup analysis of HDL-C. The degrees of interference of confounders on the relationship between serum HDL-C and SFTS mortality are displayed in the figure. The interaction between HDL-C and BMI, hypertension history and drinking history, is significant in the subgroup analysis
