# Supplementary material for: Dyslipidemia in severe fever with thrombocytopenia syndrome patients: A retrospective cohort study
Source: PLoS Negl Trop Dis. 2024 Dec 11;18(12):e0012673. doi: 10.1371/journal.pntd.0012673 (PMC11634008; doi:10.1371/journal.pntd.0012673)
Supplement: S7 Fig — (PDF) [file pntd.0012673.s012.pdf]

Variables	Survival N	Survival Mean	Survival SD	Death N	Death Mean	Death SD	HR per 1 SD	P value	P for Interaction
**Age**									0.843
≤60	129	1.74	0.62	7	1.12	0.27	0.244(0.079-0.755)	0.014*	
(60,65]	46	1.76	0.67	10	1.35	0.47	0.446(0.185-1.072)	0.071	
(65,70]	80	1.64	0.59	21	1.19	0.51	0.463(0.275-0.780)	0.004**	
(70,75]	61	1.61	0.61	15	1.21	0.34	0.462(0.240-0.886)	0.020*	
>75	49	1.55	0.67	15	1.22	0.55	0.550(0.278-1.089)	0.086	
**Gender**									0.408
Male	178	1.63	0.57	41	1.25	0.48	0.497(0.347-0.712)	<0.001***	
Female	187	1.71	0.67	27	1.17	0.41	0.331(0.185-0.590)	<0.001***	
**BMI**									0.634
≤18.5	32	1.48	0.51	7	1.36	0.45	0.783(0.328-1.866)	0.581	
(18.5,22.9]	150	1.74	0.60	25	1.32	0.47	0.441(0.268-0.724)	0.001**	
(22.9,24.9]	81	1.63	0.59	17	1.07	0.45	0.319(0.162-0.629)	<0.001***	
>24.9	102	1.66	0.71	19	1.17	0.41	0.396(0.212-0.740)	0.004**	
**Time from Onset to Admission(days)**									0.081
≤4	87	1.67	0.66	17	1.27	0.48	0.412(0.204-0.833)	0.014*	
(4,6]	125	1.64	0.61	23	1.36	0.46	0.638(0.404-1.007)	0.053	
(6,8]	104	1.55	0.60	21	1.08	0.43	0.371(0.213-0.647)	<0.001***	
(8,10]	17	1.91	0.66	5	1.11	0.31	0.131(0.020-0.857)	0.034*	
>10	32	2.07	0.51	2	0.82	0.10	0.040(0.000-8.488)	0.239	
**Hypertension History**									0.748
N	278	1.68	0.60	47	1.28	0.48	0.442(0.305-0.640)	<0.001****	
Y	87	1.63	0.71	21	1.08	0.36	0.369(0.201-0.676)	0.001**	
**Diabetes History**									0.477
N	319	1.72	0.63	58	1.25	0.46	0.427(0.309-0.592)	<0.001****	
Y	46	1.36	0.54	10	1.00	0.30	0.317(0.104-0.962)	0.042*	
**Cardiovascular Disease History**									0.887
N	340	1.66	0.61	58	1.22	0.45	0.425(0.306-0.592)	<0.001****	
Y	25	1.78	0.80	10	1.20	0.46	0.349(0.117-1.038)	0.058	
**Cerebrovascular Disease History**									0.135
N	330	1.68	0.63	60	1.26	0.46	0.446(0.322-0.616)	<0.001****	
Y	35	1.55	0.59	8	0.91	0.28	0.190(0.050-0.721)	0.015*	
**Choronic Hepatitis B History**									0.474
N	350	1.68	0.63	65	1.22	0.46	0.422(0.307-0.580)	<0.001****	
Y	15	1.54	0.53	3	1.10	0.13	0.238(0.025-2.247)	0.210	
**Suspected Hypothyroidism**									>0.999
N	342	1.67	0.63	68	1.22	0.45	0.423(0.309-0.579)	<0.001****	
Y	23	1.75	0.64	0					
**Smoking History**									0.750
N	305	1.69	0.65	54	1.20	0.45	0.395(0.275-0.570)	<0.001****	
Y	60	1.59	0.49	14	1.27	0.48	0.538(0.305-0.950)	0.032*	
**Drinking History**									0.881
N	294	1.70	0.65	53	1.23	0.45	0.411(0.281-0.600)	<0.001****	
Y	71	1.58	0.49	15	1.16	0.45	0.490(0.299-0.804)	0.005**	
**Statin History**									0.441
N	338	1.67	0.61	54	1.22	0.47	0.412(0.293-0.579)	<0.001****	
Y	27	1.66	0.83	14	1.19	0.40	0.562(0.249-1.269)	0.165	

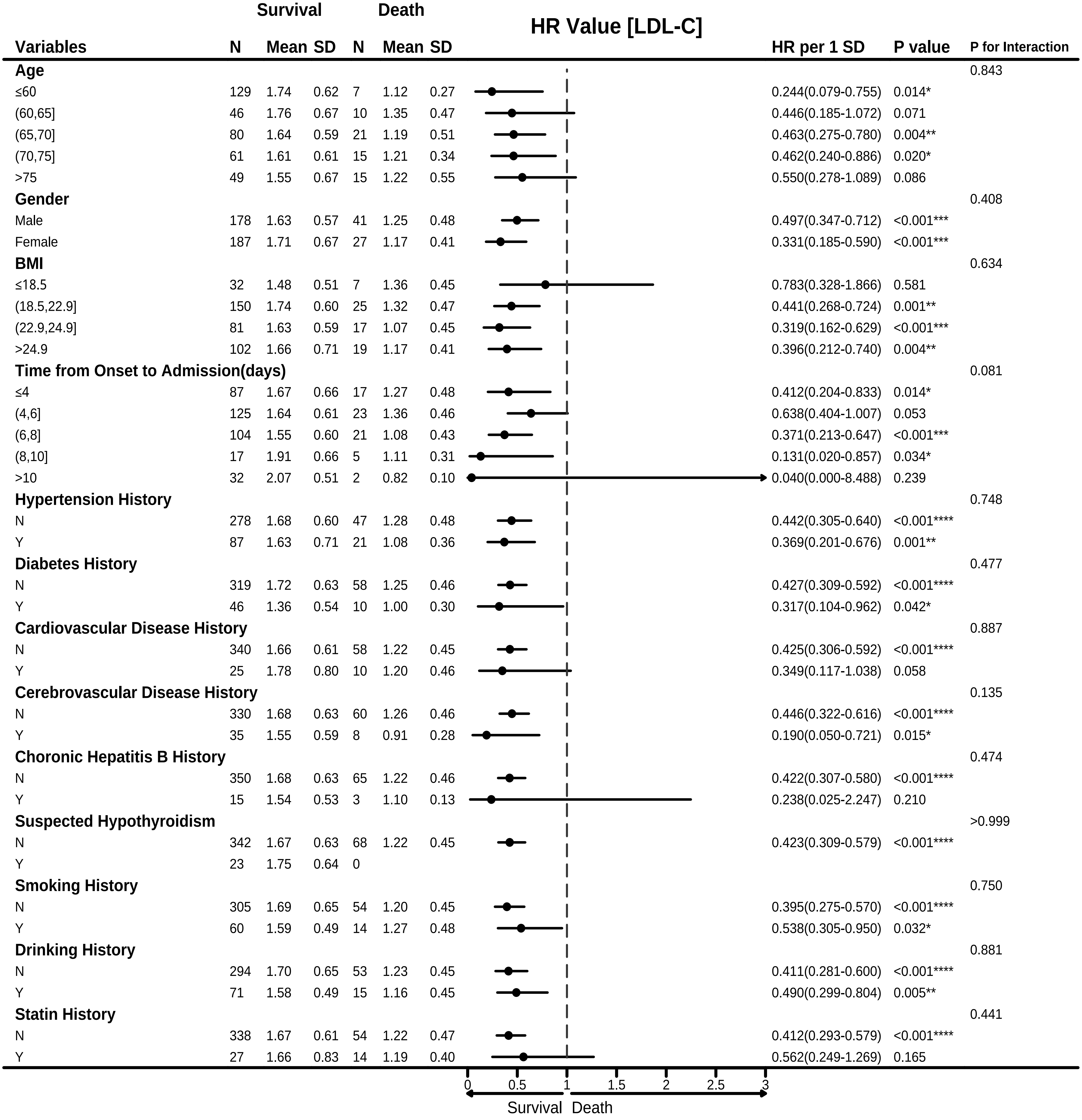

Fig S7. Subgroup analysis of LDL-C. The degrees of interference of confounders on the relationship between serum LDL-C and SFTS mortality are displayed in the figure. There are no significant interaction factors with LDL-C.
