# Supplementary material for: Dyslipidemia in severe fever with thrombocytopenia syndrome patients: A retrospective cohort study
Source: PLoS Negl Trop Dis. 2024 Dec 11;18(12):e0012673. doi: 10.1371/journal.pntd.0012673 (PMC11634008; doi:10.1371/journal.pntd.0012673)
Supplement: S8 Fig — (PDF) [file pntd.0012673.s013.pdf]

Variables	Survival N	Survival Mean	Survival SD	Death N	Death Mean	Death SD	HR per 1 SD	P value	P for Interaction
**Age**									0.817
≤60	129	149.83	177.02	7	100.00	97.13	0.659(0.214-2.028)	0.467	
(60,65]	46	127.02	148.09	10	156.00	261.69	1.108(0.645-1.905)	0.710	
(65,70]	80	119.94	133.84	21	87.90	127.34	0.759(0.419-1.375)	0.363	
(70,75]	61	97.51	137.99	15	76.87	66.98	0.794(0.367-1.718)	0.558	
>75	49	141.41	150.78	15	107.20	109.50	0.745(0.393-1.414)	0.368	
**Gender**									0.074
Male	178	133.66	149.27	41	124.83	166.21	0.929(0.674-1.280)	0.651	
Female	187	127.55	161.14	27	64.78	64.28	0.441(0.191-1.021)	0.056	
**BMI**									0.007**
≤18.5	32	113.75	130.93	7	134.86	150.66	1.149(0.582-2.268)	0.689	
(18.5,22.9]	150	141.69	166.72	25	132.72	172.47	0.949(0.624-1.444)	0.807	
(22.9,24.9]	81	122.73	161.14	17	110.12	134.87	0.831(0.508-1.358)	0.459	
>24.9	102	125.58	140.42	19	38.58	34.47	0.097(0.016-0.592)	0.012*	
**Time from Onset to Admission(days)**									0.122
≤4	87	123.49	131.95	17	99.53	112.25	0.814(0.459-1.443)	0.481	
(4,6]	125	114.89	127.24	23	144.26	201.55	1.176(0.837-1.652)	0.351	
(6,8]	104	133.38	184.16	21	64.38	55.32	0.373(0.119-1.170)	0.091	
(8,10]	17	120.24	141.50	5	81.20	93.65	0.695(0.228-2.126)	0.524	
>10	32	206.97	199.51	2	49.50	31.82	0.002(0.000-Inf)	0.487	
**Hypertension History**									0.004**
N	278	126.87	151.33	47	121.36	159.50	0.959(0.711-1.293)	0.784	
Y	87	142.21	167.68	21	55.38	43.42	0.205(0.050-0.837)	0.027*	
**Diabetes History**									0.757
N	319	133.63	159.80	58	105.26	146.64	0.785(0.572-1.076)	0.132	
Y	46	109.02	118.54	10	76.20	65.16	0.720(0.319-1.625)	0.429	
**Cardiovascular Disease History**									0.548
N	340	130.13	155.21	58	104.28	146.78	0.810(0.591-1.109)	0.189	
Y	25	136.00	159.36	10	81.90	65.73	0.641(0.255-1.612)	0.345	
**Cerebrovascular Disease History**									0.072
N	330	133.47	160.43	60	108.52	144.41	0.832(0.611-1.133)	0.242	
Y	35	102.80	90.60	8	44.50	40.95	0.244(0.044-1.343)	0.105	
**Choronic Hepatitis B History**									0.495
N	350	133.46	157.49	65	103.98	140.10	0.794(0.585-1.078)	0.139	
Y	15	62.20	58.35	3	36.00	29.46	0.556(0.106-2.924)	0.488	
**Suspected Hypothyroidism**									>0.999
N	342	132.85	156.19	68	100.99	137.74	0.776(0.571-1.054)	0.105	
Y	23	1.75	0.64	0					
**Smoking History**									0.833
N	305	129.05	153.11	54	97.07	136.14	0.777(0.543-1.112)	0.168	
Y	60	138.05	167.06	14	116.07	148.00	0.867(0.468-1.607)	0.650	
**Drinking History**									0.951
N	294	123.23	147.32	53	93.85	134.78	0.785(0.546-1.130)	0.194	
Y	71	160.73	182.74	15	126.20	149.82	0.800(0.432-1.482)	0.479	
**Statin History**									0.417
N	338	129.20	152.27	54	106.54	151.96	0.840(0.605-1.165)	0.296	
Y	27	147.22	191.62	14	79.57	54.88	0.584(0.227-1.503)	0.265	

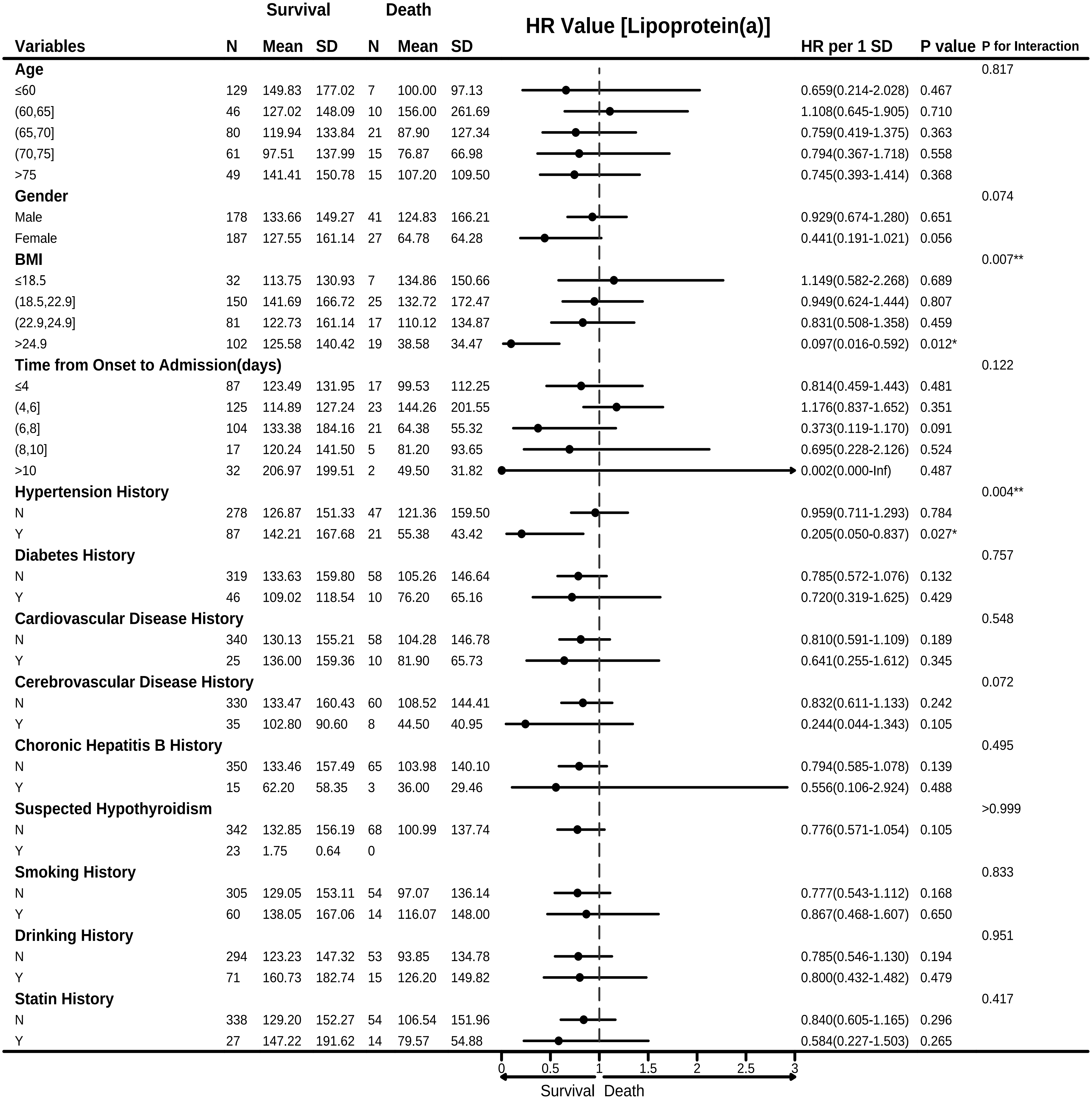

Fig S8. Subgroup analysis of lipoprotein(a). The degrees of interference of confounders on the relationship between serum lipoprotein(a) and SFTS mortality are displayed in the figure. The interaction between lipoprotein(a) and BMI and hypertension history is significant in the subgroup analysis
