# Supplementary material for: Dyslipidemia in severe fever with thrombocytopenia syndrome patients: A retrospective cohort study
Source: PLoS Negl Trop Dis. 2024 Dec 11;18(12):e0012673. doi: 10.1371/journal.pntd.0012673 (PMC11634008; doi:10.1371/journal.pntd.0012673)
Supplement: S10 Fig — (PDF) [file pntd.0012673.s015.pdf]

Variables	Survival N	Survival Mean	Survival SD	Death N	Death Mean	Death SD	HR per 1 SD	P value	P for Interaction
**Age**									0.279
≤60	129	0.75	0.23	7	0.51	0.17	0.290(0.113-0.742)	0.010**	
(60,65]	46	0.76	0.25	10	0.66	0.21	0.624(0.299-1.302)	0.209	
(65,70]	80	0.78	0.27	21	0.58	0.20	0.378(0.195-0.734)	0.004**	
(70,75]	61	0.75	0.23	15	0.61	0.21	0.582(0.345-0.980)	0.042*	
>75	49	0.70	0.27	15	0.65	0.25	0.806(0.456-1.425)	0.459	
**Gender**									0.133
Male	178	0.73	0.23	41	0.64	0.21	0.661(0.473-0.922)	0.015*	
Female	187	0.77	0.26	27	0.56	0.21	0.382(0.230-0.634)	<0.001***	
**BMI**									0.331
≤18.5	32	0.76	0.31	7	0.62	0.20	0.528(0.173-1.610)	0.262	
(18.5,22.9]	150	0.78	0.24	25	0.70	0.22	0.706(0.465-1.072)	0.103	
(22.9,24.9]	81	0.72	0.22	17	0.57	0.21	0.509(0.294-0.882)	0.016*	
>24.9	102	0.73	0.25	19	0.52	0.18	0.346(0.189-0.634)	<0.001***	
**Time from Onset to Admission(days)**									0.534
≤4	87	0.69	0.24	17	0.56	0.20	0.528(0.297-0.939)	0.030*	
(4,6]	125	0.74	0.25	23	0.67	0.18	0.749(0.472-1.188)	0.219	
(6,8]	104	0.75	0.24	21	0.57	0.25	0.436(0.253-0.752)	0.003**	
(8,10]	17	0.90	0.26	5	0.59	0.18	0.261(0.074-0.912)	0.035*	
>10	32	0.87	0.17	2	0.65	0.35	1.195(0.142-10.075)	0.870	
**Hypertension History**									0.120
N	278	0.75	0.24	47	0.64	0.23	0.623(0.450-0.863)	0.004**	
Y	87	0.77	0.26	21	0.53	0.17	0.372(0.211-0.653)	<0.001***	
**Diabetes History**									0.314
N	319	0.75	0.24	58	0.62	0.22	0.574(0.431-0.765)	<0.001***	
Y	46	0.73	0.31	10	0.51	0.15	0.276(0.092-0.828)	0.022*	
**Cardiovascular Disease History**									0.904
N	340	0.75	0.24	58	0.61	0.22	0.530(0.391-0.717)	<0.001****	
Y	25	0.78	0.28	10	0.62	0.20	0.495(0.214-1.147)	0.101	
**Cerebrovascular Disease History**									0.210
N	330	0.76	0.25	60	0.63	0.21	0.561(0.417-0.754)	<0.001***	
Y	35	0.68	0.21	8	0.46	0.16	0.349(0.141-0.867)	0.023*	
**Choronic Hepatitis B History**									0.743
N	350	0.75	0.25	65	0.61	0.21	0.533(0.401-0.710)	<0.001****	
Y	15	0.71	0.21	3	0.56	0.28	0.450(0.102-1.976)	0.290	
**Suspected Hypothyroidism**									>0.999
N	342	0.75	0.25	68	0.61	0.21	0.533(0.403-0.706)	<0.001****	
Y	23	1.75	0.64	0					
**Smoking History**									0.081
N	305	0.76	0.26	54	0.59	0.21	0.458(0.328-0.640)	<0.001****	
Y	60	0.70	0.18	14	0.69	0.23	0.979(0.570-1.680)	0.937	
**Drinking History**									0.412
N	294	0.76	0.26	53	0.60	0.21	0.484(0.347-0.673)	<0.001****	
Y	71	0.70	0.17	15	0.65	0.23	0.778(0.465-1.301)	0.338	
**Statin History**									0.713
N	338	0.75	0.24	54	0.62	0.23	0.558(0.410-0.760)	<0.001***	
Y	27	0.77	0.29	14	0.56	0.16	0.493(0.235-1.033)	0.061	

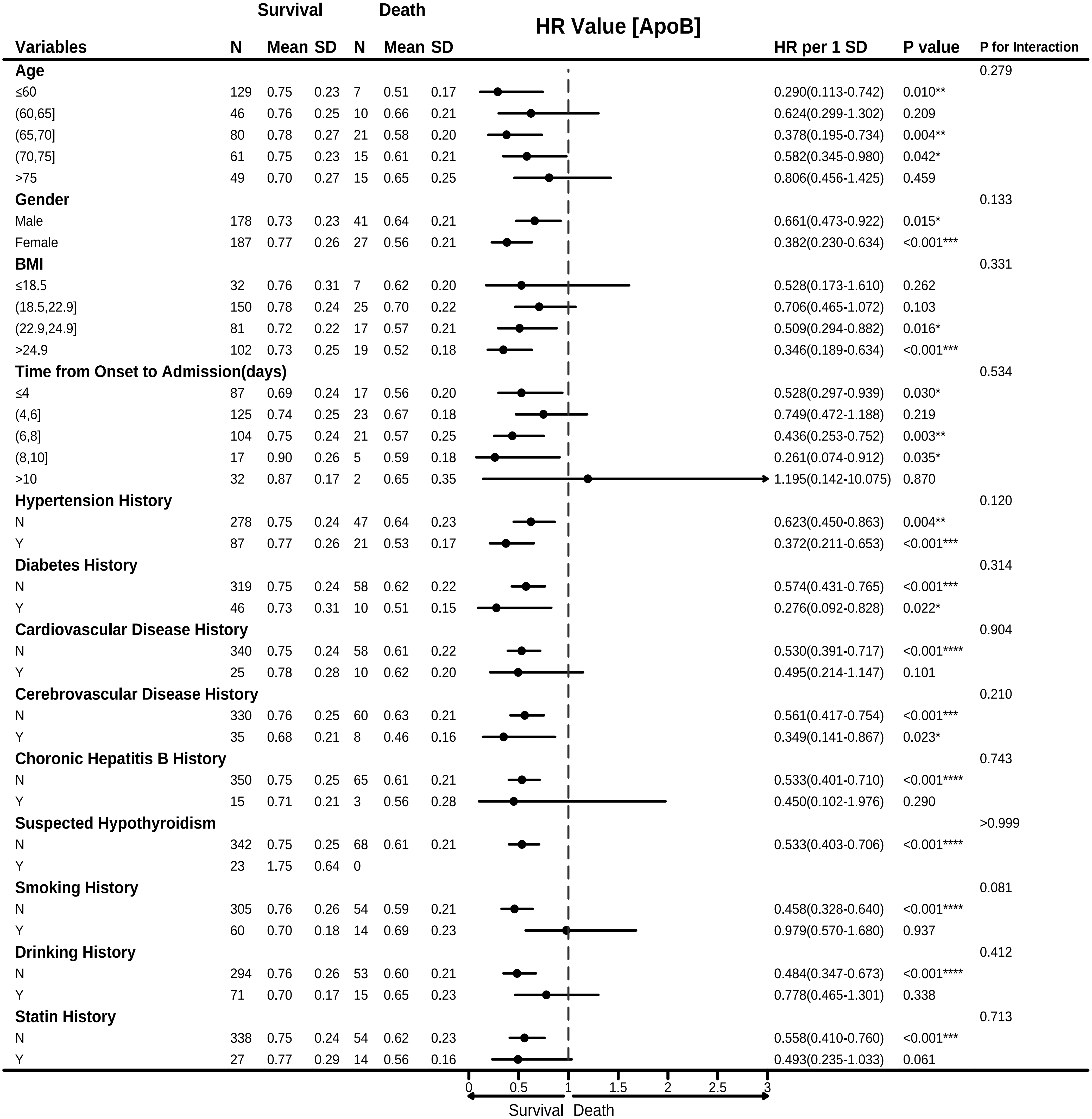

Fig S10. Subgroup analysis of ApoB. The degrees of interference of confounders on the relationship between serum ApoB and SFTS mortality are displayed in the figure. There are no significant interaction factors with ApoB.
