# Supplementary material for: Dyslipidemia in severe fever with thrombocytopenia syndrome patients: A retrospective cohort study
Source: PLoS Negl Trop Dis. 2024 Dec 11;18(12):e0012673. doi: 10.1371/journal.pntd.0012673 (PMC11634008; doi:10.1371/journal.pntd.0012673)
Supplement: S11 Fig — (PDF) [file pntd.0012673.s016.pdf]

Variables	Survival N	Survival Mean	Survival SD	Death N	Death Mean	Death SD	HR per 1 SD	P value	P for Interaction
**Age**									0.633
≤60	129	1.47	0.58	7	1.99	0.64	1.747(1.065-2.868)	0.027*	
(60,65]	46	1.51	0.67	10	1.69	0.71	1.251(0.739-2.116)	0.405	
(65,70]	80	1.43	0.48	21	1.75	0.73	1.527(1.029-2.265)	0.036*	
(70,75]	61	1.50	0.57	15	1.86	0.68	1.611(1.034-2.511)	0.035*	
>75	49	1.68	0.61	15	1.77	1.15	1.181(0.669-2.083)	0.567	
**Gender**									0.866
Male	178	1.50	0.56	41	1.76	0.83	1.432(1.088-1.884)	0.010*	
Female	187	1.50	0.59	27	1.85	0.76	1.498(1.112-2.019)	0.008**	
**BMI**									0.902
≤18.5	32	1.52	0.58	7	2.03	0.90	1.827(0.930-3.590)	0.080	
(18.5,22.9]	150	1.47	0.62	25	1.74	0.85	1.422(1.020-1.983)	0.038*	
(22.9,24.9]	81	1.53	0.58	17	1.86	0.74	1.542(1.013-2.348)	0.044*	
>24.9	102	1.50	0.50	19	1.72	0.79	1.371(0.955-1.970)	0.087	
**Time from Onset to Admission(days)**									0.524
≤4	87	1.66	0.61	17	2.00	0.84	1.546(1.009-2.368)	0.045*	
(4,6]	125	1.49	0.56	23	1.55	0.64	1.097(0.750-1.604)	0.632	
(6,8]	104	1.50	0.59	21	1.96	0.93	1.770(1.261-2.484)	<0.001***	
(8,10]	17	1.19	0.38	5	1.56	0.41	1.964(0.858-4.499)	0.110	
>10	32	1.25	0.40	2	1.63	1.20	0.047(0.000-10.842)	0.271	
**Hypertension History**									0.526
N	278	1.51	0.55	47	1.81	0.85	1.535(1.203-1.958)	<0.001***	
Y	87	1.47	0.64	21	1.75	0.70	1.360(0.962-1.922)	0.082	
**Diabetes History**									0.899
N	319	1.49	0.57	58	1.78	0.82	1.449(1.171-1.794)	<0.001***	
Y	46	1.54	0.62	10	1.86	0.67	1.527(0.888-2.628)	0.126	
**Cardiovascular Disease History**									0.306
N	340	1.50	0.58	58	1.78	0.73	1.422(1.151-1.758)	0.001**	
Y	25	1.45	0.52	10	1.87	1.18	1.841(1.045-3.241)	0.035*	
**Cerebrovascular Disease History**									0.304
N	330	1.49	0.57	60	1.74	0.78	1.392(1.120-1.730)	0.003**	
Y	35	1.53	0.58	8	2.24	0.86	1.972(1.088-3.575)	0.025*	
**Choronic Hepatitis B History**									0.495
N	350	1.50	0.57	65	1.78	0.80	1.439(1.172-1.767)	<0.001***	
Y	15	1.38	0.61	3	2.14	0.97	2.098(0.805-5.464)	0.129	
**Suspected Hypothyroidism**									>0.999
N	342	1.50	0.57	68	1.79	0.80	1.473(1.207-1.798)	<0.001***	
Y	23	1.75	0.64	0					
**Smoking History**									0.091
N	305	1.49	0.58	54	1.87	0.82	1.596(1.286-1.982)	<0.001****	
Y	60	1.52	0.55	14	1.50	0.65	0.956(0.555-1.647)	0.871	
**Drinking History**									0.126
N	294	1.50	0.59	53	1.87	0.83	1.601(1.286-1.994)	<0.001****	
Y	71	1.49	0.52	15	1.52	0.64	1.007(0.613-1.655)	0.978	
**Statin History**									0.756
N	338	1.50	0.57	54	1.81	0.87	1.502(1.204-1.875)	<0.001***	
Y	27	1.42	0.57	14	1.73	0.43	1.240(0.769-1.998)	0.378	

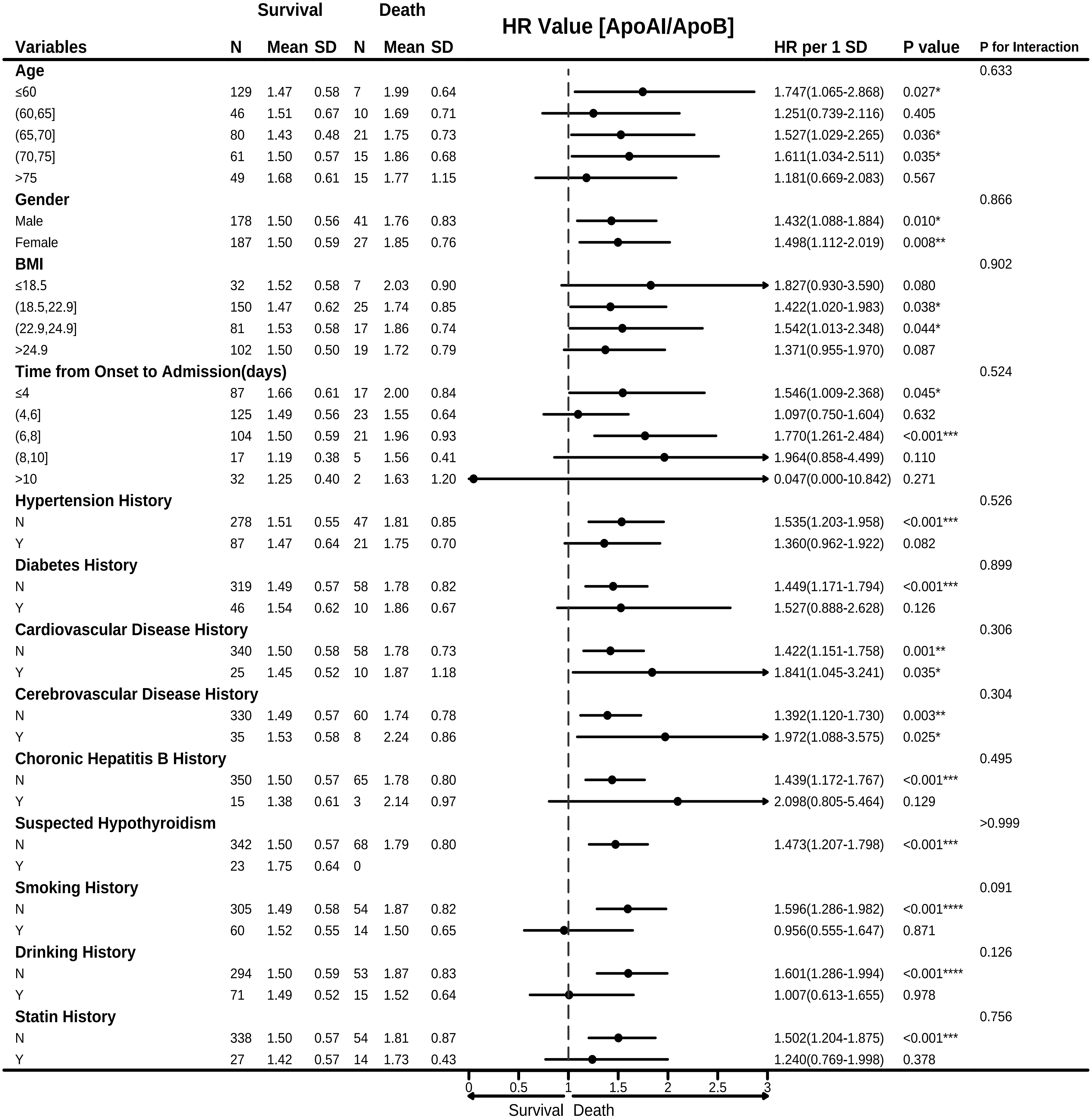

Fig S11. Subgroup analysis of ApoAI/ApoB. The degrees of interference of confounders on the relationship between serum ApoAI/ApoB and SFTS mortality are displayed in the figure. There are no significant interaction factors with ApoAI/ApoB.
